# Tracking the Near Eastern origins and European dispersal of the western house mouse

**DOI:** 10.1038/s41598-020-64939-9

**Published:** 2020-05-19

**Authors:** Thomas Cucchi, Katerina Papayianni, Sophie Cersoy, Laetitia Aznar-Cormano, Antoine Zazzo, Régis Debruyne, Rémi Berthon, Adrian Bălășescu, Alan Simmons, François Valla, Yannis Hamilakis, Fanis Mavridis, Marjan Mashkour, Jamshid Darvish, Roohollah Siahsarvi, Fereidoun Biglari, Cameron A. Petrie, Lloyd Weeks, Alireza Sardari, Sepideh Maziar, Christiane Denys, David Orton, Emma Jenkins, Melinda Zeder, Jeremy B. Searle, Greger Larson, François Bonhomme, Jean-Christophe Auffray, Jean-Denis Vigne

**Affiliations:** 1 Archéozoologie, Archéobotanique: Sociétés, Pratiques et Environnements (AASPE), UMR 7209, CNRS, Muséum national d’Histoire naturelle, Paris, France; 2 Malcolm H. Wiener Laboratory for Archaeological Science, American School of Classical Studies, Souidias 54, 10676 Athens, Greece; 3 Centre de Recherche sur la Conservation (CRC), Muséum national d’Histoire naturelle, CNRS, Ministère de la Culture, CP 21, 36 rue Geoffroy Saint-Hilaire, 75005 Paris, France; 4 Centre de recherche en Paléontologie Paris, UMR7207, Muséum national d’Histoire naturelle, CNRS, Sorbonne Université, 8 rue Buffon, 75005 Paris, France; 5 DGD-REVE, Muséum national d’Histoire naturelle, 17 Place du Trocadéro, bureau E205, 75016 Paris, France; 6 Vasile Pârvan, Institute of Archaeology, Romanian Academy, 11 Henri Coandă Street, Bucarest, Romania; 7 Department of Anthropology, University of Nevada, Las Vegas/Desert Research Institute, Reno, Nevada USA; 8 Archéologies et Sciences de l’Antiquité (Arscan), UMR 7041 CNRS, Université de Paris Nanterre, Paris I, 92023 Nanterre, France; 9 Joukowsky Institute for Archaeology and the Ancient World, Brown University, Box 1837, 60 George Street, Providence, RI 02912 USA; 10 Ephorate of Palaeoanthropology and Speleology, Hellenic Ministry of Culture and Sports, Ardittou 34B, 11636 Athens, Greece; 11 Department of Biology, Faculty of Sciences, Ferdowsi University of Mashhad, Mashhad, Iran; 12 Paleolithic Department, National Museum of Iran, Tehran, Iran; 13 Department of Archaeology and Anthropology, University of Cambridge, Downing Street, Cambridge, CB2 3DZ UK; 14 Archaeology, School of HASS, University of New England, Armidale, NSW 2351 Australia; 15 Research Institute of Cultural Heritage and Tourism (RICHT), Iranian Center for Archaeological Research (ICAR), Tehran, Iran; 16 Near Eastern Archaeology, Institute für Archäologie Wissenschaften, Johann Wolfgang Goethe Universität, Frankfurt am Main, Germany; 17 Institut de Systématique, Evolution, Biodiversité (ISYEB), UMR 7205, Muséum national d’Histoire naturelle, Sorbonne Université, Ecole Pratique des Hautes Etudes, Université des Antilles, CNRS, Paris, France; 18 BioArCh, Department of Archaeology, University of York, York, YO10 5DD UK; 19 Institute for the Modelling of Socio-Environmental Transitions, Bournemouth University, Talbot Campus, Poole, BH12 5BB UK; 20 Department of Anthropology, National Museum of Natural History, Smithsonian Institution, Washington, District of Columbia USA; 21 Department of Ecology and Evolutionary Biology, Corson Hall, Cornell University, Ithaca NY, 14853-2701 USA; 22 Palaeogenomics and Bio-Archaeology Research Network, School of Archaeology, University of Oxford, Oxford, OX1 3TG UK; 23 Institut des Sciences de l’Evolution (ISEM), UMR 4554, CNRS, IRD, EPHE, Université de Montpellier, Montpellier, France

**Keywords:** Biochemistry, Computational biology and bioinformatics, Ecology, Molecular biology, Zoology

## Abstract

The house mouse (*Mus musculus*) represents the extreme of globalization of invasive mammals. However, the timing and basis of its origin and early phases of dispersal remain poorly documented. To track its synanthropisation and subsequent invasive spread during the develoment of complex human societies, we analyzed 829 *Mus* specimens from 43 archaeological contexts in Southwestern Asia and Southeastern Europe, between 40,000 and 3,000 cal. BP, combining geometric morphometrics numerical taxonomy, ancient mitochondrial DNA and direct radiocarbon dating. We found that large late hunter-gatherer sedentary settlements in the Levant, c. 14,500 cal. BP, promoted the commensal behaviour of the house mouse, which probably led the commensal pathway to cat domestication. House mouse invasive spread was then fostered through the emergence of agriculture throughout the Near East 12,000 years ago. Stowaway transport of house mice to Cyprus can be inferred as early as 10,800 years ago. However, the house mouse invasion of Europe did not happen until the development of proto urbanism and exchange networks — 6,500 years ago in Eastern Europe and 4000 years ago in Southern Europe — which in turn may have driven the first human mediated dispersal of cats in Europe.

## Introduction

The impact of our species on biodiversity was initiated with the global dispersal of *Homo sapiens* from the Late Pleistocene^1–3^. Anthropogenic biological invasions are another key component of biodiversity loss^4^. Despite their natural occurrence since the beginning of life on Earth, biological invasions have drastically increased with human activities^5^, facilitating species dispersal^6^. The house mouse (*Mus musculus* ssp.) is emblematic of these anthropogenic biological invasions threatening biodiversity^7,8^. Although often overlooked compared with commensal rats (*Rattus rattus, R. norvegicus* and *R. exulans*), this elusive mammal has been a much more successful invasive rodent, becoming almost as ubiquitous as *H. sapiens*^9^. Originating in the Indo-Pakistan subcontinent and neighbouring Afghanistan and Iran^10–12^, house mice differentiated during the Pleistocene climatic oscillations^9^ into three main *Mus musculus* subspecies (*M. m. domesticus, M. m. musculus* and *M. m. castaneus*). All these subspecies are human commensals, facilitating their long-distance colonization and ultimately their cosmopolitan range^13^.

The inextricable link between human dispersal, its associated processes of niche construction and the global invasive process of the house mouse makes it a relevant bio-indicator of human impact on biodiversity, which we can track in the bioarchaeological record. Currently, bioarchaeological evidence and genetic studies on modern populations agree on the origin of *M. m. domesticus* commensal behavior associated with the Neolithic transition in the Near East^9,14^. But whether sedentism^15^ and/or the rise of the farming economy^14,16^ were the key driving factors that led to this behavioural shift is debated. The dispersal of *M. m. domesticus* towards Europe has been deemed to follow the Neolithic diaspora stemming from Southwest Asia; yet, current understanding of the zooarchaeological occurrences rather suggests a house mouse dispersal in Mediterranean Europe along with Iron Age demographic and commercial movements^17^. If this process is well documented for the western Mediterranean, it remains to be ascertained for the Eastern Mediterranean, where archaeological evidence is too scarce to discard potential earlier Neolithic or Bronze Age dispersals. Finally, the dispersal history of *M. m. musculus*, the other commensal house mouse in Europe, currently present in central and Northern Europe, is so far only documented in Chalcolithic Romania 6,500 years ago^14,18^, but its origin and the timing of its dispersal route need to be unravelled.

The commensal house mouse is considered to have initiated the commensal trajectory of the cat towards domestication^19–22^, implying that tracking the bioarchaeological history of the former lays the trail for the latter. Mitochondrial DNA suggests that the domestication of the African wild cat (*Felis silvestris lybica*) took place amidst the rise of agriculture in the Neolithic Near East^23,24^. The earliest and most striking evidence of cat domestication comes from 9,500 cal BP in Pre-Pottery Neolithic (PPN) Cyprus^19,25^. Its introduction onto the island is thought to be tied to the control of the proliferation of the house mouse populations, present on the island since the Early PPNB^26^. The appearance of the domestic cat in western European archaeological contexts during the Iron Age, around 3,000 years ago, is synchronous with the strong evidence for the house mouse biological invasion of western Europe^17^. This co-dispersal of cats and house mice has also been mentioned in literary sources, describing the deliberate transport of domestic cats on ships to control rodent pests, inducing its worldwide distribution^24,27^. This co-phylogeography supports the premise that understanding the house mouse’s origin and dispersal can lead to insights pertaining to the origin of domestic cats and their subsequent dispersal.

To document the timing and pace of human mediated house mouse dispersal and provide new insights into the origin and dispersal of domestic cats, we collected and analysed 829 ancient mouse (*Mus* sp.) dental remains from 43 archaeological sites located in Southwest Asia and Europe, spanning 40,000 years from the Upper Pleistocene to the Late Bronze Age. Their numerical taxonomy was performed using geometric morphometric (GMM) analyses of the first lower molar (m1) shape as a proxy^28^, using Bayesian models and machine learning approaches. In addition, 85 samples for ancient mtDNA sequences (Cytochrome *b*) and direct radiocarbon dating were collected on the GMM identified specimens to support the taxonomic identification and provide direct dates respectively.

## Results

### Strategy for data acquisition

**Figure 1:**
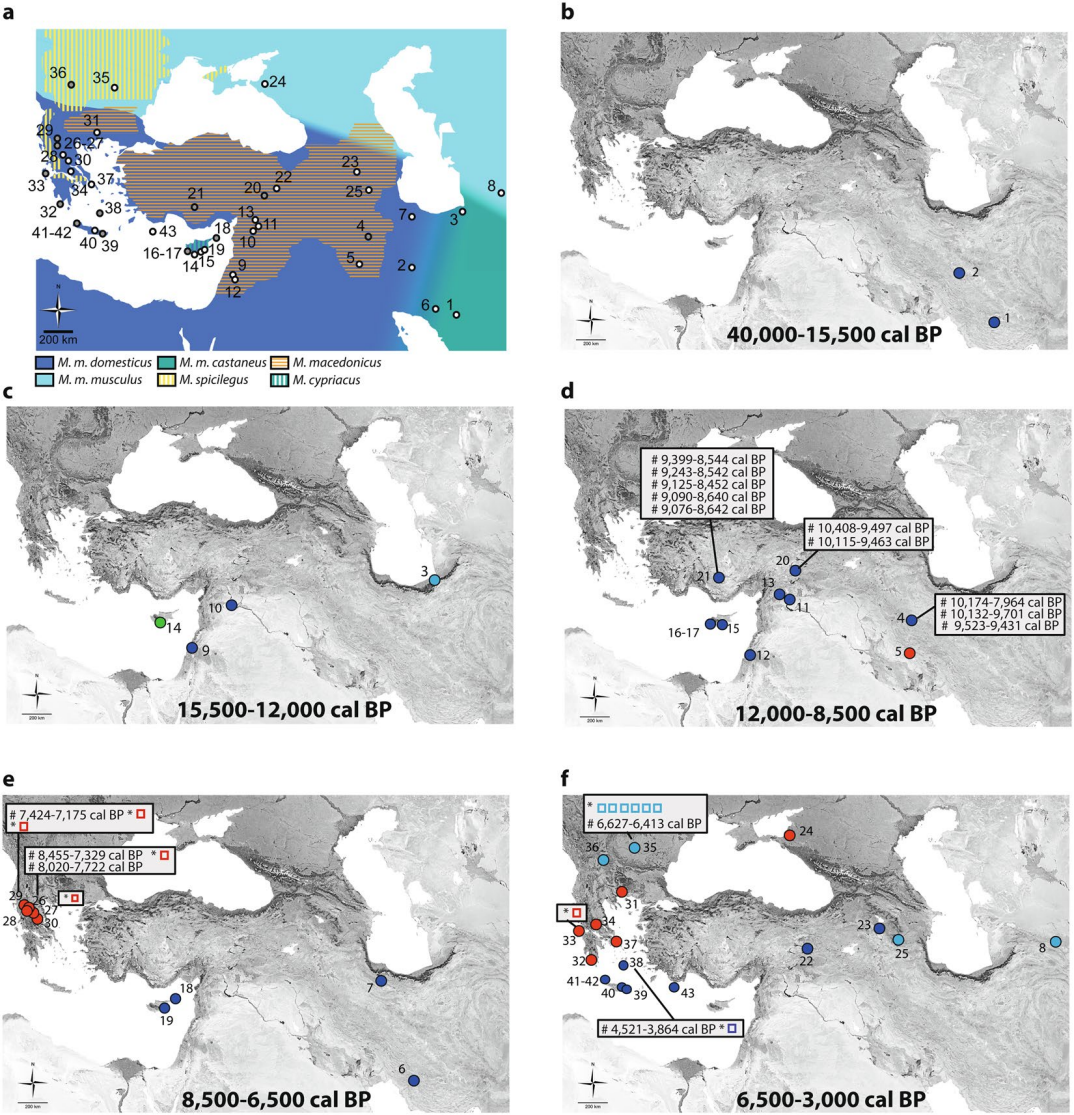
Spatio-temporal representation of the house mouse dispersal in Southwestern Asia and Eastern Europe. (**a**) Current distribution of the three commensal subspecies from the studied area: *M. m. domesticus*, *M. m. musculus*, *M. m. castaneus* and the three non-commensal species: *M. spicilegus*, *M. macedonicus* and *M. cypriacus*. Localisation of the archaeological sites studied. Circles filled in grey identify sites where aDNA and radiocarbon analyses have been performed (See the supplementary note S2 for details). 1: Qaleh Bozi, 2: Eskaft-e Gavi, 3: Ali Tappeh, 4: Ganj Dareh, 5: Ali Kosh, 6: Tol-e Nourabad, 7: Tepe Zagheh, 8: Ulug Depe, 9: ‘Ain Mallaha, 10: Mureybet, 11: Jerf El Ahmar, 12: Netiv Hagdud, 13: Dja’dé, 14: Akrotiri-Aetokremnos, 15: Klimonas, 16: Kissonerga-Mylouthkia 1, 17: Kissonerga-Mylouthkia 2, 18: Cape Andreas-Kastros, 19: Khirokitia, 20: Cafer Höyük, 21: Çatalhöyük, 22: Norsun Tepe, 23: Ovçular Tepesi, 24: Chishko, 25: Kohne Pasgah Tepesi, 26: Mavropigi, 27: Xirolimni, 28: Theopetra, 29: Avgi, 30: Koutroulou Magoula, 31: Dikili Tash, 32: Alepotrypa, 33: Drakaina, 34: Sarakenos, 35: Bucșani La Pod, 36: Vinča-Belo Brdo, 37: Ayia Triada, 38: Akrotiri, 39: Mochlos, 40: Malia, 41: GSE, 42: Chania, 43: Uluburun shipwreck (See supplementary Note S2 for details). (**b–f**) Diachronic mapping of the dispersal of the *M. musculus* lineages identified by numerical taxonomy performed using GMM on the first lower molar: *M. m. domesticus* (deep blue full circle), *M. m. musculus* (light blue full circle), *M. spicilegus/M. macedonicus* (red full circle) and *M. cypriacus* (green full circle). Direct radiocarbon dating at 95.4% probability (#) and the ancient mtDNA Cytochrome *b* taxonomic identification (*) are provided (See the Supplementary Table S6 and S12 for details). Each square represents a specific aDNA identification and its color corresponds to three taxonomic units: *M. m. domesticus* (deep blue square), *M. m. musculus* (light blue square), and *M. spicilegus*/*M. macedonicus* (red square). Map adapted from (https://d-maps.com) by D.G. Kuriyama. Figure generated by TC and KP in Adobe Illustrator CS6.

Our *Mus* sp. archaeological dataset includes 829 specimens from 43 sites sampled across the Middle East and Eastern Mediterranean Europe, as a core area for the origin and spread of agriculture in Europe, close to the cradle of *Mus musculus* ssp. lineages, along a temporal span from 40,000 cal BP to 3,000 cal BP (Fig. 1b location, Supplementary Table S1). We defined five key chronological phases of human history in the studied area: (1) the pre-sedentism period: 40,000–15,500 cal BP, (2) the early sedentary communities of hunter-gatherers: 15,500–12,000 cal BP, (3) the early agrarian economy and dispersal in the Near East and Cyprus: 12,000–8,500 cal BP, (4) the Neolithic dispersal towards Europe: 8,500–6,500 cal BP, and (5) the Late Neolithic/Bronze Age exchange and trade networks: 6,500–3,000 cal BP.

Samples came from *in situ* deposits of *Mus* sp. remains, excluding specimens from disturbed archaeological contexts. Some sites provided only one specimen which was kept for analysis due to the reliability of their original context (Supplementary note S2). The GMM numerical taxonomy of the archaeological specimens relies on a comparative analysis of 512 genotyped specimens (Supplementary Table S3) including the three wild species (*M. macedonicus, M. spicilegus, M. cypriacus*) and three commensal subspecies (*M. m. domesticus, M. m. musculus* and *M. m. castaneus*) present in the studied area. We assessed the phenotypic diversity and relationships among the modern and archaeological “populations” using Bayesian models and machine learning classification methods, in order to build a diachronic map of the emergence and dispersal of the commensal subspecies across the five chronological phases (See *Methods* section).

Among the 829 specimens selected for GMM identification, only 85 were suitable for an integrated approach combining GMM, aDNA and radiocarbon dating from the same specimen. Indeed, this analytical approach necessitated complete mandibles which could be divided into three sub-samples: the m1 tooth for the GMM analysis and half the hemi-mandible for aDNA and radiocarbon dating. Considering the size and the weight range of half hemi-mandibles samples (from 6 to 40 mg), this approach proved challenging (See *Methods* section). Among the 85 samples selected, only 17 provided positive results for radiocarbon dating and only 15 genuine ancient sequences for the Cytochrome *b* fragment (Supplementary Table S4).

### Pre-Neolithic mice in Southwestern Asia

We identified *M. m. domesticus* in the Iranian Plateau from two Middle and Upper Paleolithic cave deposits in the central and southern Zagros (Fig. 1b, Supplementary Methods S5). These *Mus* remains from Qaleh Bozi and Eskaft-e Gavi result from non-anthropogenic deposits likely accumulated by birds of prey^29–31^, providing the oldest fossil evidence for the presence of non-commensal *M. m. domesticus* populations in the Iranian Plateau before the Neolithisation process in the Near East.

At the transition between the Pleistocene and the Holocene, we found *M. m. domesticus* in the earliest sedentary open-air settlements of hunter-gatherers in the Southern and the Northern Levant (Fig. 1c, Supplementary Methods S5), from the early Natufian layers of ‘Ain Mallaha between 14,500 and 13,000 cal BP and from the later Natufian and Khiamian layers of Mureybet at 12,000 cal BP (Fig. 1c), respectively.

The occurrence of *M. m. musculus* in the Ali Tappeh cave between 15,201–14,758 and 12,080–11,615 cal BP (Fig. 1c, SI Fig. 1) provides the evidence for this subspecies in Northeastern Iran during the Late Glacial. However, we cannot relate this occurrence with an *in situ* commensal accumulation, since archaeological evidence does not support any sedentary occupation of the cave by human communities^32,33^.

In the Epipaleolithic Akrotiri-Aetokremnos rock-shelter in Cyprus (Fig. 1c, Supplementary Methods S5), from where the earliest human presence on Cyprus has been inferred^34,35^, we found evidence of *Mus cypriacus*, the extant endemic mouse of Cyprus^36^. This confirms its presence on the island before the human Neolithic colonization, and probably since the Middle Pleistocene, considering the phenotypic relationship of the fossil *Mus* remains in Cape Pyla^37,38^ with *Mus cypriacus*^39^.

### Pre-Pottery Neolithic (PPN) range expansion in Southwestern Asia

We found *M. m. domesticus* populations in the early PPNA farming villages of the Northern Levant (Jerf El Ahmar) and Southern Levant (Netiv Hagdud), dated to 12,000 cal BP (Fig. 1d, Supplementary Methods S5) as well as between 11,100 and 10,600 cal BP in the PPNA village of Klimonas, Cyprus (Fig. 1d, Supplementary Methods S5). This identification in Klimonas relies on a single molar found in a secure context within the floor deposit of the village’s communal building, but its occurrence in greater numbers is supported by 41 remains of *Mus* sp. and gnawing marks on suid bones^19,40^. These clues suggest that with early farmers of the Levantine PPNA culture, came the earliest evidence of house mouse stowaway transport onto an island. Our results confirm the presence of *M. m. domesticus* in large numbers during the PPNB colonization of Cyprus (Kissonerga-Mylouthkia) up to 8,000 cal BP in Khirokitia, on the southern coast of Cyprus, and in Cape Andreas-Kastros, on the northeastern tip of the Khyrenia Peninsula, indicating the successful settlement of commensal populations of *M. m. domesticus* throughout the island (Fig. 1d, Supplementary Methods S5).

Between 10,000 and 8,000 cal BP, *M. m. domesticus* spread inland from the Levantine cradle towards the upper Euphrates valley in the Taurus foothills (Çafer Höyük), southern Zagros (Ganj Dareh) and the Konya Plain in Anatolia (Çatalhöyük) (Fig. 1e, Supplementary Methods S5). Such inland occurrence has been directly dated (Supplementary Table S6) for Çafer Höyük (between 10,408 and 9,275 cal BP), Ganj Dareh (between 10,174 and 9,431 cal BP) and Çatalhöyük (between 9,399 and 8,452 cal BP).

### House mouse dispersal outside the PPN core area

Northward from the PPN core area, we found evidence for the Late Neolithic and Chalcolithic dispersal of *M. m. domesticus* towards Transcaucasia in Norsun Tepe and Ovçular Tepesi, between 5,000 and 4,000 cal BP (Fig. 1f, S5 Fig. 1, Supplementary Methods S5), supporting the role of the Near East in the Neolithic makeup of Transcaucasia^28^. Eastward, we found the presence of *M. m. domesticus* in the southern Zagros (Tol-e Nourabad) and Iranian Plateau (Tepe Zagheh) between 7,000 and 6,000 cal BP (Fig. 1e, Supplementary Methods S5), which could be due to a local commensalism process rather than the consequence of a dispersal event from the Levant. This suggestion is supported by the occurrence of *M. m. domesticus* on the Iranian Plateau since at least the Middle Pleistocene; the mitochondrial genetic distance between Iranian and Near Eastern *M. m. domesticus* populations^41^; and the genomic divide between the Neolithic human populations of Anatolia and the Zagros region^42^.

Westward, towards the Eastern Mediterranean and continental Southeastern Europe, we have no evidence of a Neolithic dispersal of *M. m. domesticus* beyond Cyprus (Fig. 1f, Supplementary Methods S5). All of the ten samples from Early, Middle and Late Neolithic contexts from continental Greece have been identified as the autochthonous “wild” phenotype (*Mus macedonicus*) with GMM and Cytochrome *b* (Supplementary Table S6) and directly dated at Mavropigi (8,455 - 7,329 cal BP) and Avgi (7,424 - 7,175 cal BP). These results support the absence of a maritime Neolithic dispersal of house mouse towards the Eastern Mediterranean and continental Southeastern Europe further west than Cyprus during the 11-10^th^ millennia cal BP. Secondly, they show that the Neolithic spread through the southern Aegean islands and Northern Greece during the 9^th^ millennium cal BP^43–45^ did not act as a vector of house mouse dispersal towards Southeastern Europe.

In Aegean contexts, the occurrence of *M. m. domesticus* is only documented from the Bronze Age (Fig. 1f, SI Fig. 1), where it occurs in all the Early, Middle and Late Bronze Age contexts of urban sites in Crete (GSE, Chania, Mochlos, and Malia) and Santorini (Akrotiri), strongly supporting the absence of house mouse in Neolithic contexts^17^. Its occurrence in Akrotiri is confirmed by the Cytochrome *b* identification dated between 4521 and 3,864 cal BP (Supplementary Table S6). This ubiquity of *M. m. domesticus* in all the Aegean Bronze Age contexts of our dataset suggests that the invasive process of *domesticus* in the Eastern Mediterranean was driven mainly by Bronze Age maritime networks, which is directly confirmed by the house mouse mandible found in the cargo of the Late Bronze Age Uluburun shipwreck off the southern shores of Anatolia^46^.

Although we could have expected *M. m. domesticus* to first invade the European continent, fostered by the Neolithic and Bronze Age connectivity with the Near East, we found instead that *M. m. musculus* was the first house mouse to spread into continental southern Europe at the end of the Neolithic (Fig. 1f, Supplementary Methods S5). Its occurrence is documented from Late Neolithic / Chalcolithic household deposits (mid 7^th^ millennium BP) from tell sites in Southeastern Romania (Bucșani) and Serbia (Vinča-Belo Brdo). Its identification has been confirmed in Bucșani by ancient Cytochrome *b* sequences, secured for six specimens and a direct radiocarbon dating between 6,627 and 6,413 cal BP (Supplementary Table S6). The *M. m. musculus* remains in Vinča-Belo Brdo have not been directly dated but they have been sampled from a deposit that derives from a fire event confidently dated to 6510–6460 cal BP^47^. The occurrence of commensal *musculus* has also been documented in Turkmenistan by 3,000 cal BP, with the remains of *musculus* being found in a storage jar from the proto-urban tell site of Ulug Depe (Fig. 1f, Supplementary Methods S5).

## Discussion

### Upper Pleistocene range expansion of house mice in Southwest Asia

Speciation models from modern mitochondrial markers suggest that the range expansion of *M. m. domesticus* in the Mesopotamian area took place at some point during the Middle Pleistocene^48^. Our study found occurrence of *M. m. domesticus* in the Upper Pleistocene ecosystem of the southwestern Iranian Plateau^29^,confirming a long-lasting natural occurrence of these lineages in this area^11,12^. Yet, we only found occurrence of *M. m. domesticus* in the Southern Levant from 14,500 cal BP, and in Northern Levant from 12,000 cal BP. In addition, its absence from numerous Middle and Upper Pleistocene non-anthropogenic cave deposits in the Southern Levant^15,39^ suggests that the natural range expansion of *M. m. domesticus* which took place in the ecological gradient of the Euphrates-Tigris River Basin, only reached the Levant after the Last Glacial Maximum^49^, in keeping with the phylogeographic reconstruction derived from mitochondrial data^41^ and its probable dating^48^. A suitable climatic refuge that could have occurred in the relatively lower altitude areas of the Euphrates-Tigris River Basin should be investigated.

### The origin of house mouse synanthropy

The earliest commensal populations of *M. m. domesticus* found in Natufian sedentary settlements (14,500 cal BP) confirm that the impact of sedentism on ecosystems and the ecology of organisms (i.e. reduction of predation and competition pressures, climatic buffer etc^15,50,51^) was the catalyst for the commensal relationship between mice and humans rather than the emergence of agriculture systems with large-scale grain storage^14,16^, which emerged two millennia later. Nevertheless, *M. m. domesticus* was identified only in the largest, long-term Natufian settlements such as ‘Ain Mallaha in the Southern Levant and Mureybet in the Northern Levant between 14,500 and 12,000 BP. In smaller and shorter term Natufian sites in the Southern Levant, only the native mouse *Mus macedonicus* was identified^15,17,39^. This pattern suggests that dense human occupation in large open air settlements was the prerequisite for *M. m. domesticus* to eventually outcompete other potential anthropophilous rodent like *M. macedonicus* from the Natufian ecological niche^15^.

The occurrence of *M. m. domesticus* in all the PPNA and Early and Late PPNB contexts of our dataset in the Zagros, Levant, Anatolia, and Cyprus, suggests that the emergence of the agricultural system was the key driving force in the house mouse’s commensal trajectory. PPNA plant cultivation of wild cereals and pulses^52–54^ correlates with the emergence of the first settlement with communal buildings and cereal storage^55,56^, marking a substantial increase in the degree of sedentism of human societies. PPNB plant and animal domestication, which entails a greater reliance on cereals^57^ and storage^14,58^, correlates with an increase in settlement sizes from less than two hectares, during the PPNA, to more than 10 hectares during the Late PPNB^16,59^. All these factors prompted the development of proto-urban environments with a unique anthropogenic ecosystem disturbance^60^, fostering the subsequent adaptedness of the house mouse to such altered human environments^61^. With regards to agriculture, the villages and buildings provided greater protection against predators and competitors, a buffer from temperature fluctuation, and a constant food supply due to large scale grain storage from the PPNA^53,56^ onwards, driving *M. m. domesticus* to become an anthro-dependent organism^62^.

### House mouse dispersal in Southeastern Europe

After a steady dispersal throughout the PPN core area, including Cyprus, from two potential commensal epicentres in the Northern and Southern Levant, the house mouse dispersal did not follow the spread of Neolithic culture towards Europe through the Aegean islands or the Bosphorus. According to our current data, the dispersal of *M. m. domesticus* outside the PPN core area did not reach the Aegean before the Bronze Age (4,000 years ago), suggesting that dispersal barriers might have prevented its biological invasion of the Aegean and continental Europe along with the Neolithic dispersal of domestic animals and plants^63^.

The biological invasion model of *M. m. domesticus* towards the Aegean and continental Greece could be understood according to a “mainland-island” metapopulation structure^64^, based on long distance dispersal from source populations in the PPN core area. To be successful during the Neolithic, this biological invasion would have required a sustainable local environment for house mouse metapopulations to thrive and disperse, as well as a migrant flow from the source to maintain them. To explain the absence of house mouse Neolithic dispersal outside the PPN core area, we consider that neither the ecological niche nor the migrant flow could sustain this biological invasion model. Before about 7,000 BP, most of the Neolithic communities in Greece and Northern Balkans lived in small settlements lacking communal storage facilities^58,65–67^. Furthermore, our study proved that indigenous rodents such as *Mus macedonicus* or *M. spicilegus*, occupied the commensal niche in the south Balkan peninsula at least, acting as a competitive barrier. Only the intensification of maritime trade with the Near East driven by Bronze Age cities^43,68^ and the increasing size and stability of settlements associated with this migrant flow could have sustained *M. m. domesticus* metapopulations in the Aegean and the Balkan peninsula^69^.

The earliest house mouse dispersal in Europe was achieved by *M. m. musculus*, colonizing Eastern Europe at the end of the Neolithic, 6,500 years ago. Our understanding of the origin of *M. m. musculus* subspecies from phylogeographic studies is still very limited^9^. From the different debated points of origin of this subspecies, in the southern or northern Caucasus^10,70,71^, a human dispersal with the advance of agriculture into Europe through the Pontic Steppe north of the Black Sea has been considered the most parsimonious^9^. However this phylogeographic scenario does not fit with the archaeological understanding of the agricultural dispersal considered to enter into Europe through the Balkans and then reaching the Pontic steppes from the western Black Sea shores between the 7^th^ and 6^th^ millennium BP^72^. Our current dataset cannot rule out a dispersal route of *M. m. musculus* along the southern Black Sea coasts and through the Bosphorus. However, a natural expansion range of *M. m. musculus* into the Pontic steppe through Transcaucasia or Turkmenistan is likely to have happened from the Late Glacial warm up. Indeed, unlike *M. m. domesticus*, this sub-species has greater non-commensal abilities that could have allowed it to spread in the Pontic steppes without any facilitation from the human niche construction. Then, when the Neolithisation reached the Pontic steppes from the west by the 7^th^ millennium BP, the Neolithic settlements could act as a commensalism center for *musculus*, much like the PPN niche construction in the Levant did for *M. m. domesticus*. This assumption needs to be demonstrated through investigation of small mammal remains from early Neolithic settlements in Ukraine to document its commensalism, as well as through a comparison with late Pleistocene/early Holocene small mammal remains documenting the Pontic biodiversity before the Neolithic. Then, the development of large proto-urban centers, such as the large Tripolye settlements from the Dnieper river to the Carpathians, stemming from a broader context of large late-Neolithic settlements and the exchange networks stretching from eastern Croatia to Ukraine, could have both facilitated the dispersal of *M. m. musculus* metapopulations reaching as far as southeastern Romania (Bucșani) and Serbia (Vinča-Belo Brdo).

### Could the house mouse dispersal scenario help elucidate early cat domestication?

The cat domestication process was historically thought to have been initiated alongside the development of agriculture 6,000 years ago in the Nile valley, when mouse proliferation in villages attracted commensal populations of small felids^21,73^. However, research on the last 12,000 years of human impact on Cyprus’ vertebrate diversity has provided insights into a greater time depth for the human–cat relationship. Cyprus is an oceanic island which has remained remote from the continent since the Miocene^74^. Its endemic fauna, which included only one carnivorous species, a genet (*Genetta plesictoides*), was exctinct before the arrival of Neolithic settlers 11,000 years ago; except for the endemic Cypriot mouse (*Mus cypriacus*) that is still extant today^36^. Zooarchaeological evidence from the PPNA site of Klimonas showed that early Neolithic colonisers introduced specimens of *Felis silvestris* cf. *lybica* to Cyprus at least 11,000 years ago^40^. This human dispersal of small felids, acting likely as commensals control, is associated with the earliest evidence for the presence of *M. m. domesticus* in Cyprus (as shown by our study) and with the earliest cereal cultivation on the island^75^. The earliest evidence for domestic cat in Cyprus, however, comes 9,000 years ago at the PPNB site of Shillourokambos. Here, a complete skeleton of *Felis silvestris lybica*^25^ 18% bigger than its wild relatives in Cyprus and on the continent^19^, was found in association with a human burial^25^, suggesting a tight relationship between the deceased and the cat^25^. The human dispersal in Cyprus of human-controlled individuals of *Felis silvestris* cf. *lybica* 11,000 years ago, potentially to control house mouse pests, and the occurrence of domestic specimens of *Felis silvestris lybica* from 9,000 BP, strongly suggest that the cat domestication process was already underway in the Levant, at least five millennia before the earliest evidence of cat domestication in Egypt^19^.

Since cats are strictly carnivorous, evolved for preying on small terrestrial vertebrates and birds, it is widely accepted that humans have taken advantage of these phenotypic traits to control rodent pests in their grain storage and to hunt birds^19^. For these reasons it is widely assumed that the commensal pathway of cat domestication has been triggered by the presence of commensal populations of rodents within human dwellings^27^. Therefore our identification of many commensal *M. m. domesticus* in the Early Natufian village of ‘Ain Mallaha could provide an indirect clue for a cat commensal pathway initiated as early as 14,500 BP in the Southern Levant, 4,000 years before the beginning of agriculture. This hypothesis of opportunistic commensal small felids in the vicinity of Natufian sedentary settlements is supported by the scarce occurrence of *Felis silvestris* ssp. remains that were collected from Natufian and Khiamian levels at Hatoula^76^, Mureybet^77^ and the Late Natufian site of Ein Gev II^78^. Unidentified *Felis* remains in the Natufian site of ‘Ain Mallaha^79^ and Iraq ed-Dubb in Jordan^80^ have also been recorded. To support this hypothesis, greater efforts need to be undertaken in the taxonomic identification of small felid remains associated with the Natufian settlements of the Levant, combining the latest advances in morphometric, proteomic or genomic approaches where possible.

Paleogenetic studies provide clear evidence that the first human mediated dispersal of *F. s. lybica* towards Europe stemmed from Anatolia, spreading towards current Bulgaria by 6,400 cal BP, Romania by 5,200 cal BP^24^ and up to Poland by 5,000 cal BP^81^. Yet, archaeological evidence from Kastanas (3,300 cal BP) suggests that cat dispersal only reached continental Greece during the Late Bronze Age^24,82^. As cats are strict carnivores specialised in rodent predation and because they were mostly appreciated by humans for fighting against murid pests^20,21,83^, it is very likely that the anthropogenic dispersal of domestic *F. s. lybica* to Europe during the Late Neolithic / Chalcolithic was driven by the need for rodent pest control in the Balkan peninsula. Therefore, the timing of the invasive process of house mice in Europe that we have documented here can provide elements of understanding for the tempo of these two cat dispersal pulses into Southeastern Europe.

We propose as a hypothesis that the earliest cat dispersal towards Europe was driven by *M. m. musculus* biological invasion during the Late Neolithic/Chalcolithic, when the size of the proto-urban settlements and the catchment for grain production generated rodent pests and therefore the need to tackle them with cat predation. The later dispersal in continental Greece on the other hand could have been pushed by the later *M. m. domesticus* dispersal associated with the development of Bronze Age urbanisation and the need for pest control in the Balkan peninsula. House mouse is indeed documented on Crete from this study in the port cities of Mochlos^84^, Malia and Chania by 3,900–3,700 cal BP and in Kommos during the same period^85^. It reached continental Greece later as suggested by its earliest occurence at the site of Nichoria in the Peloponnese^17^. The presence of cats οn Crete by the Late Bronze Age is supported by various iconographic representations, such as the reliefs of miniature cats on vessel bodies also found in the port towns of Malia^86^, which could indicate that the Cretans had known or acquired cats through their established connections with Egypt^87^. To support this hypothesis of two domestic cat dispersal routes out of Southwest Asia, at the end of the Neolithic in the Balkans and during the Bronze Age in the Greek peninsula, an extensive survey for cat remains associated with direct radiocarbon dating and high-throughput paleogenetics analyses to capture recent phylogeographic lineages within *F.s.lybica* needs to be pursued.

## Conclusion

The bioarchaeological evidence generated here has revealed new insights into the origin of the biological invasion of the two house mouse sub-species in Southwestern Asia and Europe as well as indirect clues on the earliest steps of cat domestication and dispersal towards Europe. They suggest a natural range expansion of *M. m. domesticus* in the ecological gradient of the Tigris-Euphrates Basin up to the eastern Mediterranean coasts during the Late Pleistocene, stemming from the Zagros. In the Levant, sedentism of Natufian communities created the ecological niche that promoted the commensal behaviour of *M. m. domesticus* populations from 14,500 BP, and potentially the commensal pathway of cat domestication. However, this synanthropic behaviour was restricted to large and densely populated proto-village environments and potentially happened independently in the Southern and Northern Levant. It is only with the advance of the PPN agricultural ecosystem and its proto-urban environments, denser human populations and greater human movements, that the biological invasion by *M. m. domesticus* happened in the Near East and up to the remote island of Cyprus, where the house mouse, and human-controlled commensal cats, followed the earliest maritime transport of the PPNA ecosystem by 10,800 BP.

Curiously, the Neolithic dispersal stemming from the Near East along the Mediterranean and the Bosphorus routes did not foster the house mouse invasion of Europe. The first invasive wave by *M. m. musculus* happened during the Late Neolithic / Chalcolithic, when it reached south-eastern Europe by 6,500 BP. After a natural range expansion into the Pontic Steppes from the Late Glacial, *M. m. musculus* likely became commensal when Neolithic settlements reached the Pontic steppes from Southeastern Europe by 7,000 BP. Then, the inland dispersal of *M. m. musculus* into Europe was facilitated by human movements along transport and exchange networks. The second invasive wave by *M. m. domesticus* only penetrated the Aegean by 4,000 BP when the intensification of Bronze Age maritime trade with the Near East and the emergence of urban environments fostered sustainable metapopulation structures. This invasive process by the two commensal house mice and their pressure on grain storage of farming communities could have significantly contributed to the first dispersal waves of domestic cats into Europe.

The models of origin and dispersal for the two house mouse sub-species in Southwestern Asia and Europe generated by this study need to be further tested using high-throughput sequencing and paleogenomic approaches. Retrieving mitochondrial haplotypes from ancient house mouse will produce dated phylogeographic inferences about the colonization history of both sub-species in Europe and indirect clues about past human dispersal and trading networks. Genome-wide studies of ancient commensal populations across Southwestern Asia and along a time series from the Late Glacial to the Iron Age will potentially allow the investigation of (1) the genetic signatures for the behavioural selection involved in the commensalism process, (2) specific phenotypic traits separating *M. musculus* sub-species from other wild species dwelling around the Mediterranean, such as the tail length, longer in *M. musculus* sub-species and (3) the amount of genetic isolation and introgression with autochtonous *Mus* species involved in the evolutionary process of the house mouse.

## Methods

### Geometric morphometrics

The acquisition of GMM data for the molar shape analysis of the first lower molars (m1) was performed on 2D images of the occlusal view of the m1, following an already published protocol^28^. The images were acquired by a Leica EZ4D stereoscope digital camera and the LAS operating software. The 2D external outline of the occlusal view of the m1 was recorded using tpsDig v. 2.30^88^.

The phenotypic similarities between the current *Mus* sibling species^89^ (Fig. 1a) forced us to find the best GMM method to capture the taxonomic signal from the m1 outline by comparing the classification performances of four mathematical representations and three approaches of the dental outline measurements. The four mathematical representations are two semi-landmark alignment^90^ methods (Bending Energy Minimization (BEM) and Procrustes Distance Projection (PDP)) and two Elliptic Fourier^91^ methods (Procrustes Aligned Elliptic Fourier (EFAproc) and Normalized Elliptic Fourier (NEF)^92^). The three methods for the m1 outline data acquisition were: one landmark and 63 semi-landmarks^15,28^, six landmarks on the cusps maximum of curvature and 52 semi-landmarks on the rest of the curves, five landmarks on the maximum of curvature between the cusps (valleys) and 48 semi-landmarks. The taxonomic performance of each approach was assessed over a training set of 30 *M. m. domesticus*, 30 *M. m. musculus*, and 20 *M. macedonicus* specimens. We used the classification method from the Linear Discriminant Analysis (LDA) including a leave-one-out cross validation on a reduced shape dataset^90,93^ using R libraries (*MASS, Momocs, Geomorph, shapes)* and R functions (see^92^). The results suggested that the BEM methods applied on outlines with 1 landmark and 63 semi-landmarks to be the most efficient geometric morphometric approach in capturing the taxonomic signal required for this study (Supplementary Table S7). The BEM method was therefore applied for the whole dataset.

To assess the numerical taxonomy of the archaeological samples we performed the analysis in several steps. First, the potential occurrence of sympatric wild and commensal subspecies in each of the 43 archaeological deposits was explored using a Bayesian model based clustering approach^94^, to avoid pooling two taxa within the same “population” grouping factor (i.e. site name) (Supplementary Table S8). Then we assessed the phenotypic differences and affinities among the modern and archaeological “population” samples using MANOVA and Canonical Variate Analysis (CVA), after dimensionality reduction^90,93^ using R library MASS^95^. And finally, to identify the taxonomic status of each archaeological “population” mean shape in the discriminant morphospace (CV1–CV2), we used the machine learning k-nearest neighbours (KNN) algorithm using four taxonomic units as training vectors: the three *M. musculus* subspecies and one non-commensal group including *M. macedonicus*, *M. spicilegus* and *M. cypriacus*. The number of *k* values was determined by the square root of *N*. KNN machine learning was performed using R library Class^95,96^.

### Paleogenetic analysis

Samples for ancient DNA analyses consisted mostly of the extracted molars from the hemi-mandibles used for paleogenetic and radiocarbon dating analyses. DNA extractions and analyses of ancient samples were performed in a dedicated clean room facility at the Musée de l’Homme (Plateau de Paléogénomique et Génétique Moléculaire, MNHN) where no previous work on either modern or ancient mouse DNA has been performed. Modern and standard experiments were performed in the Service de Systématique Moléculaire (MNHN, Paris). The total amount of tooth material per specimen ranged from 2 up to 12 mg (mean 4.5 mg). For several specimens some bone material was used instead of teeth (range 10–21.5 mg, mean 17.5 mg). DNA extractions were performed using the PrepFiler BTA Forensic DNA Kit (Life Technologies) according to the manufacturer’s recommendations (with a final elution volume of 35 µl). Lysis was performed via the direct digestion of complete extracted teeth (without prior crushing).

We investigated the amplifiability of mouse DNA through absolute quantification using qPCR (CFX-96 real-time thermal Cycler, Bio-Rad technologies) and a series of three nested PCR fragments of the cytochrome *b* gene: 65, 92 and 133 bp (Supplementary Table S9). Despite being less variable than the more documented mitochondrial D-loop region, the cytochrome *b* gene allows the design of PCR primers that enable the amplification of very short fragments (the hypervariable region of the control region spans more than 300 bp in mice which prevents such design^48^). Furthermore, Suzuki *et al*.^97,98^ have shown that the overall phylogenetic signal is congruent between the mitochondrial D-loop and the cytochrome *b* gene, with the same five main haplogroups being recognized within *M. musculus*. We designed the qPCR primers based on formerly published material for the cytochrome *b* gene from both house mouse and wild mice species^97^. All primer pairs excluded human amplification thanks to numerous mismatches in priming sites (Supplementary Table S10), even when the PCR reactions were spiked with up to 10 µg of human DNA. We produced a quantitation standard (using a modern mouse sample as a template DNA for the 133 bp fragment) to address the sensitivity of the assay (from 1 million down to 1 copy per µl of extract): all three assays were optimized for the same annealing temperature and sensitized down to 2–5 copies per µl (Supplementary Table S11). We designed these assays in order to maximize their discrimination power at two taxonomic scales. Firstly, they allow for a strict diagnosis of each of the three wild species (*M. spicilegus*, *M. macedonicus* and *M. cypriacus*) versus the commensal forms (Supplementary Table S12). Secondly, we could discriminate between the known modern haplogroups of *M. m. musculus* and *M. m. domesticus* thanks to 1, 2, and 3 fixed positions in the three nested amplicons respectively (Supplementary Table S12). PCR reactions were carried out in 25 µl using 1X SsoAdvanced Supermix (Bio-Rad Technologies), 200 µM of each forward and reverse primer (Supplementary Table S11), and 1 µl of DNA extract. Reaction conditions were as follows: 2 min initial denaturation at 95 °C, then 40 cycles of 10 s denaturation at 95 °C, 15 s at 58 °C and 20 s at 72 °C. PCR results are summarized in Supplementary Table S11.

DNA sequencing of the positive PCR products were performed on a 3130 ABi automated sequencer with BigDye v1.1, using extended sequencing primers to allow for the sequence determination of short amplicons^99^. Only haplotypes validated through at least two independent PCRs were considered in the manuscript (see Supplementary Table S11 for details).

### AMS dating

Collagen extraction of the samples weighed between 6.5 and 36.9 mg, followed the protocol of Cersoy *et al*.^100^. For this study we shortened the duration of the bone demineralization and the collagen purification to minimize collagen degradation or loss. The quality control parameters (%C, %N, and C/N ratios) reported here were measured in this EA and did not require an extra sample to be taken.

Depending on collagen weight, samples were either graphitized or transferred to the gaseous phase (CO_2_). Heavy samples (> 0.2 mgC) were combusted and graphitized using an AGE3 device (Ion plus, Switzerland)^101^. In order to reduce the risk of memory effects in the graphite reactors, a sample of about the same age was combusted prior to each archaeological sample. Ultra-light samples (<0.2 mgC) were combusted and graphitized online using a GIS device^102,103^. To improve the accuracy of the measurement in the gaseous phase (usually lower than for the graphitized samples), samples MT 58, MT 61, MT 78 and MT 81 were measured in duplicates.

All the *Mus* sp. samples were dated using the compact AMS ECHoMICADAS^104^. Data reduction was performed using BATS software (version 4.07)^105^. Oxalic acid II NIST standard and phthalic anhydride blanks were measured, for each individual run, to allow normalization, correction for fractionation and background corrections. Intercomparison bone samples (VIRI F, VIRI I VIRI H and VIRI E – for further details see^106^ – spanning the full range of radiocarbon were also prepared and radiocarbon dated. The radiocarbon ages were calibrated using OxCal software^107,108^.

The results are reported in Supplementary Table S6. Collagen yields ranged between 0.8 and 9.1% and are indicative of poor to moderately well-preserved bones. C/N ratios ranged between 3.1 and 3.6, within the 2.9–3.6 limits suitable for radiocarbon dating^109^. Carbon content [C] of the collagen extracts varied widely, between 0.012 to 1.057 mgC. Samples that were dated twice provided similar ages, allowing us to use the R_Combine function in OxCal to reduce the uncertainty of the calibrated interval. Radiocarbon ages of three intercomparison samples were in good agreement with the consensus values^106^.

## Supplementary information

Supplementary information.

Supplementary information2.

Supplementary information3.

Supplementary information4.

Supplementary information5.

Supplementary information6.

Supplementary information7.

Supplementary information8.

Supplementary information9.

Supplementary information10.

Supplementary information11.

Supplementary information12.

Supplementary information13.

## Data Availability

Full morphometric dataset to support the finding of this article can be found online at: https://datadryad.org/stash/share/W68uF68hDZ8fEDUIR8Psb-6dIZmBbh1UagYAa18o9Jw GenBank Number association for the successful ancient DNA sequences have been included in Supplementary Tables S11 and S12.
