# Supplementary material for: Tracking the Near Eastern origins and European dispersal of the western house mouse
Source: Sci Rep. 2020 May 19;10:8276. doi: 10.1038/s41598-020-64939-9 (PMC7237409; doi:10.1038/s41598-020-64939-9)
Supplement: Supplementary file 1 — Supplementary information. [file 41598_2020_64939_MOESM1_ESM.docx]

**Tracking the Near Eastern origins and European dispersal of the western house mouse**

Thomas Cucchi^1*^, Katerina Papayianni^1,2^, Sophie Cersoy^3^, Laetitia Aznar-Cormano^4^, Antoine Zazzo^1^, Régis Debruyne^5^, Rémi Berthon^1^, Adrian Bălășescu^6^, Alan Simmons^7^, François Valla^8^, Yannis Hamilakis^9^, Fanis Mavridis^10^, Marjan Mashkour^1^, Jamshid Darvish^11^†, Roohollah Siahsarvi^11^, Fereidoun Biglari^12^, Cameron A. Petrie^13^, Lloyd Weeks^14^, Alireza Sardari^15^, Sepideh Maziar^16^, Christiane Denys^17^, David Orton^18^, Emma Jenkins^19^, Melinda Zeder ^20^, Jeremy B. Searle ^21^, Greger Larson ^22^, François Bonhomme^23^, Jean-Christophe Auffray^23^, Jean-Denis Vigne^1^

^*^ Corresponding author: [cucchi@mnhn.fr](mailto:cucchi@mnhn.fr)

† deceased

^1^ Archéozoologie, Archéobotanique: Sociétés, Pratiques et Environnements, UMR 7209, CNRS, Muséum national d'Histoire naturelle, Paris, France.

^2^ Malcolm H. Wiener Laboratory for Archaeological Science, American School of Classical Studies, Souidias 54, 10676 Athens, Greece.

^3^ Centre de Recherche sur la Conservation (CRC), Muséum national d'Histoire naturelle, CNRS, Ministère de la Culture FR, 36 rue Geoﬀroy Saint-Hilaire, 75005 Paris, France

^4^ Centre de recherche en Paléontologie Paris, UMR7207 Muséum national d'Histoire naturelle, CNRS, Sorbonne Université, 8 rue Buffon 75005 Paris

^5^ DGD-REVE, Muséum national d'Histoire naturelle, 17 Place du Trocadéro, bureau E205, 75016 Paris

^6^ Vasile Pârvan, Institute of Archaeology, Romanian Academy, 11 Henri Coandă Street, Bucarest, Romania

^7^ Dept of Anthropology, University of Nevada, Las Vegas/Desert Research Institute, Reno, Nevada

^8^ Archéologies et Sciences de l’Antiquité, UMR 7041 CNRS, Université de Paris Nanterre, Paris I, 92023 Nanterre, France.

^9^ Joukowsky Institute for Archaeology and the Ancient World, Brown University, Box 1837, 60 George Street, Providence, RI 02912, USA .

^10^ Ephorate of Palaeoanthropology and Speleology, Hellenic Ministry of Culture and Sports, Ardittou 34B, 11636 Athens, Greece.

^11^ Department of Biology, Faculty of Sciences, Ferdowsi University of Mashhad, Mashhad, Iran

^12^  Center for Paleolithic Research, National Museum of Iran, Tehran, Iran.

^13^ Department of Archaeology and Anthropology, University of Cambridge, Downing Street, Cambridge CB2 3DZ, UK.

^14^ Archaeology School of HASS, University of New England, Armidale, NSW 2351 Australia.

^15^ Research Institute of Cultural Heritage and Tourism (RICHT), Iranian Center for Archaeological Research (ICAR), Tehran, Iran.

^16^ Near Eastern Archaeology, Institute für Archäologie Wissenschaften, Johann Wolfgang Goethe Universität, Frankfurt am Main

^17^ Institut de Systématique, Evolution, Biodiversité, ISYEB - ISYEB, UMR7205, Muséum national d'Histoire Naturelle, CNRS,IRD,UA,SU, Paris, France

^18^ BioArCh, Department of Archaeology, University of York, York YO10 5DD, UK

^19^ Institute for the Modelling of Socio-Environmental Transitions, Bournemouth University, Talbot Campus, Poole, BH12 5BB, UK

^20^ Department of Anthropology, National Museum of Natural History, Smithsonian Institution, Washington, District of Columbia, USA

^21^ Department of Ecology and Evolutionary Biology, Corson Hall, Cornell University, Ithaca, NY 14853-2701, USA

^22^ Palaeogenomics and Bio-Archaeology Research Network, School of Archaeology, University of Oxford, Oxford OX1 3TG, UK

^23^ Institut des Sciences de l’Evolution, ISEM—UMR 4554, CNRS, IRD, EPHE, Université de Montpellier, Montpellier, France
