# Supplementary material for: Tracking the Near Eastern origins and European dispersal of the western house mouse
Source: Sci Rep. 2020 May 19;10:8276. doi: 10.1038/s41598-020-64939-9 (PMC7237409; doi:10.1038/s41598-020-64939-9)
Supplement: Supplementary file 3 — Supplementary information3. [file 41598_2020_64939_MOESM3_ESM.docx]

**Supplementary Note S2: Description of the archaeological sites studied**

**Middle and Upper Paleolithic-Epipaleolithic-Natufian**

The earliest sites included in the project are caves, *Qaleh Bozi* *2* (QB2) and *Eshkaft-e Gavi* in Iran. QB2 is interpreted as a late Middle Paleolithic base camp which was used for consecutive months every year as a secondary consumption site; the lithic industry is of the Mousterian techno-complex; the evidence supports the hunting of local wild game (gazelles, wild sheep and goats, cervids and suids, equids and aurochs) but there is no evidence for primary sedentary habits (Biglari et al. 2009 and 2015; Jaubert et al. 2010). The sole *Mus* molar from QB2 was retrieved by water sieving with mesh sizes of 2x2 mm, and is of controversial stratigraphic security as it might be the result of an eagle owl pellet (Biglari et al. 2009; Jaubert et al. 2010).

In *Eshkaft-e Gavi* cave, lithic industries dated at the Middle/Upper Paleolithic and early Epipaleolithic were excavated; the two *Mus* specimens come from the Upper Paleolithic layers (Rosenberg 1985). The preliminary faunal data of Eskaft-e Gavi (*Gazella, Ovis, Capra, Bos, Equus*, possibly *Cervus*) do not support an argument for sedentism in either the area or the site (Rosenberg 1985). The two *Mus* molars included in this study were retrieved by soil sieving using an undefined mesh size (Scott and Marean 2009).

*Ali Tappeh* is an Epipaleolithic cave in northeast Iran, whose inhabitants practiced the extensive hunting of gazelles, goats/sheep, aurochs, jackals/foxes, seals, horses, pigs, oxen and deer during five separate chronological stages between 15.940–13.630 and 13.042–10.795 cal BP (McBurney 1969; Manca et al. 2018). The nature of this community is thought to be nomadic. There is no published evidence as to whether the pigs, oxen, sheep and goat from Ali Tappeh are of the domestic, pre-domestic or wild form; we can only assume their wild status based on the nomadic character of the community. Nevertheless, we cannot correlate this Epipaleolithic site with the Natufian sites in terms of evidence for sedentism. The two *Mus* molars included in this study were recovered via soil sieving in meshes of 0.1 and 0.3 inches (McBurney 1969); they come from Phase 1 (sounding A, decapage 24a) and Phase 2 (sounding A, decapage 10). The rodent assemblage was studied recently by Louis Arbez (2018) for his Master’s thesis under the supervision of M. Mashkour and E. Stoetzel at the Natural History Museum, Paris.

*Ain Mallaha* (*Eynan*) is an open-air settlement comprising layers that span the Middle Paleolithic, Early Epipaleolithic and Natufian period. The latter is dated between 14.495-13.967 and 12.690-12.167 cal BP and subdivided into three occupational phases (Valla et al. 2017). The Natufian inhabitants constructed semi-subterranean dwellings that included hearths and display evidence of the existence of floors and roofs. The architectural remains, the material culture uncovered and the inhumations found under the dwellings all suggest the sedentary behavior of a hunting-gathering society, considered as a precursor to the emergence of agriculture (Valla et al. 2017). Hunted game, such as boar, hares, gazelles and foxes, as well as birds, crustaceans, fish, mollusks, almonds and pistachios contributed to the diet (Valla et al. 2017; Bridault et al. 2008). The *Mus* molars included in this study were excavated from well-stratified contexts of the Early Natufian (packed floors of structures, pits and burials) and retrieved via water sieving (Weissbrod et al. 2017, supplementary information).

*Mureybet* is a tell site in the middle Euphrates valley, which was inhabited during the Late Natufian, Khiamian, PPNA and PPNB periods; the Khiamian layers are characterized by stone buildings, which were initially subterranean then subsequently above ground; they were replaced by rectangular buildings during the PPNA-B. Hearths and ovens were usually located outside the buildings. Grain was stored in both domestic and communal storage areas and even in specialized buildings. The inhabitants of Mureybet hunted gazelles, equids, aurochs, wild boar, fallow deer, wild sheep and birds; they also harvested the wild forms of barley, einkorn and rye (Ibáñez 2008a and 2008b). Mureybet is one of the key sites in the evolution of agriculture in the Fertile Crescent, and especially for the Euphrates valley, for which it preserves the eponymous culture of the PPNA: the Mureybetian (Ibáñez 2008a). The two *Mus* molars included in this study were retrieved via dry soil sieving and correspond to the Natufian 1A and the Khiamian layers dated at 12.529-11.325 and 11.604-11.209 cal BP respectively (Cucchi 2005, 135).

*Akrotiri-Aetokremnos* is an Epipaleolithic rock-shelter on coastal southwest Cyprus, which bears evidence for the earliest human presence of Epipaleolithic foraging populations on Cyprus; furthermore, numerous remains of local endemic fauna, namely pygmy elephants and hippopotami, have been unearthed. There is currently a lot of controversy in the literature regarding the correlation of humans and the endemic fauna and how much the former have facilitated the extinction of the latter (for a summary see Simmons 2013). The stratigraphy of the site includes three layers: the oldest, stratum 4, is dated around 12.791-12.682 and 12.539-12.092 cal BP and comprises many elephant bones, pygmy hippopotamus bones, a few wild boar bones and a small amount of lithic artefacts; the sterile stratum 3 lies on top of it; the cultural stratum 2 lies on top of stratum 3, and contained lithics of a continental cultural affinity associated with bird bones, shellfish, wild boar bones and some pygmy hippo bones, all pinpointing to an Epipaleolithic community dated to around 12.000 cal BP (Simmons 1999; Vigne et al. 2009). The Akrotiri-Aetokremnos *Mus* molar was retrieved after water sieving the soil of ‘Feature 10’ of Epipaleolithic 2A layer (Cucchi 2005, 137).

**PPNA Sites**

*Jerf el Ahmar* is a PPNA/early PPNB settlement dated at 11.600-10.789 and 11.237-10.757 cal BP (Stordeur et al. 1997), located on the left bank of Euphrates, whose main characteristics are the large subterranean communal buildings, around which the local society had created its reality. The older communal buildings were compartmentalized and versatile towards multi-use, either for storage or some common activities, perhaps of a symbolic function; the younger ones were simpler without interior divisions, possessing only a large bench against the wall. The communal buildings were usually burnt on purpose. The individual buildings were generally circular in the early stages of the village, evolving to rectangular as the centuries advanced. They usually surrounded the communal buildings, perhaps reflecting some early form of construction planning (Stordeur 2015). The subsistence of the villagers was based on game (onagers, hemiones, aurochs, gazelles, hare, foxes and birds are only some of the hunted species) and wild plant exploitation (wild almonds, wild barley, prunes and pistachios) (Stordeur et al. 1997). Only dogs appear in a domestic status. The sole *Mus* molar included in this study was recovered by dry sieving the soil from the interior of the houses; the mesh size remains undefined (Cucchi 2005, 135).

*Netiv Hagdud* is a PPNA settlement in the lower Jordan valley dated between 12.068-11.135 and 11.163-10.248 cal BP; the village was probably inhabited for a minimum of nine months per year. The inhabitants used oval and circular buildings and storage facilities (bins and silos), hunted gazelles, ibexes, deer, hare, boar and trapped migratory aquatic birds; they gathered wild figs, almonds, acorns and pistachios and practiced a mono-culture of wild barley (Bar-Yosef et al. 1991). The intra-settlement organization included households, public activity and waste disposal areas (as above, IBID). The *Mus* molars included in this study were retrieved after systematic wet and dry sieving and soil flotation (as above, IBID).

*Ganj Dareh* is a PPNA tell settlement in the central Zagros mountain range. Two occupation phases have been excavated: the earliest one, without any evidence for built architecture, comprised only small or large fire pits containing charcoal, stones and other materials; the later phase is characterized by varied architectural remains (Smith 1990). This profound architectural difference probably reflects two different modes of habitation: a limited seasonal occupation from spring to fall during the earlier phase and a longer sedentary one during all seasons in the later phase (as above, IBID). Subsistence was based on hunting (gazelles, red deer, hare, fox, partridge) as well as herding goats, sheep, cattle and pigs; the plant input in the diet included two-row barley, lentils, pistachios, almonds, field peas and milk vetch (as above, IBID). The Ganj Dareh goat population has been the subject of extensive research about its domestic status. Indeed, it has proven to be one the earliest wild goat populations to have received human management, marking the initial steps of the transition from hunting to herding; it can thus be referred to as domestic (Zeder and Hesse 2000). There is no information on the sieving process; the mice teeth originate probably from layer B, the chronology of which ranges between 10.217-9.912 and 9.888-9.550 cal BP, according to fossil goat ^14^C dated bones (Zeder and Hesse 2000).

*Klimonas* is a PPNA settlement in south Cyprus. In terms of space organization, a collective large subterranean circular building marks the settlement, similar to the late PPNA ones of the northern Levant (Jerf el Ahmar, Mureybet), indicating the complexity of the society (Vigne et al. 2012 and 2017). Smaller individual rounded buildings with hearths and activity areas cluster around it, either adjacent or above it. The villagers were hunting the endemic wild boar, previously mentioned at Akrotiri-Aetokremnos, and were trapping birds, freshwater turtles and freshwater crabs; they were accompanied by cats and dogs imported from the mainland. Also imported were the exploited cereals, namely emmer wheat; the domestic status of which is still undetermined; other plant food resources include pistachios, prunes and acorns (Vigne et al. 2012 and 2017). The sole *Mus* molar included in this study is the result of extensive water sieving and flotation of soils from the collective building, corresponding to dates between 11.145-10.742 and 11.055-10.523 cal BP (Vigne et al. 2012 and 2017).

**PPNB/C sites**

*Ali Kosh* is a PPNB/C tell settlement in southwest Iran which has provided evidence for the early stages of goat domestication and herd management (Zeder 2011). The site was used for a relatively short period of time, some 500 years, between 9.742-9.307 and 9.076-9.006 cal BP (Zeder and Hesse 2000). The excavations brought to light evidence for the consumption of local plants, wild legumes, wheat and barley - the latter an indication of early cereal domestication - as well as a single hornless sheep skull, further evidence of domestication (Hole et al. 1969). The faunal repertoire also comprised gazelle, onager, wild oxen, domestic cattle, pig, dog, fox, hyena, wild cat, marten, weasel, hedgehog, gerbil, rat and mouse; various birds, reptiles, fish and invertebrates were also recovered (Flannery in Hole et al. 1969). The Ali Kosh mouse mandible is thought by its excavators to be intrusive (Flannery in Hole et al. 1969, p. 319).

*Dja’dé* is a tell site on the left bank of the Middle Euphrates valley; the uncovered layers date to the end of the PPNA and mostly to the PPNB (10.650-10.199 to 10.513-9.960 cal BP). The early PPNB buildings are rectangular, replacing the PPNA circular ones; large open spaces with combustion structures were discovered between the buildings (Coqueniot 1998). The inhabitants managed wild wheat and barley in fields near the settlement and collected wild almonds, pistachios and lentils (Willcox 1996). There is evidence for an initial domestication stage of cattle, sheep, goat and pig; the inhabitants hunted several wild species, mainly gazelles, aurochs, wild equids and aquatic birds (Coqueniot 1998; Helmer et al. 2005; Vigne 2002).The 6 *Mus* molars included in this study were retrieved through the dry soil sieving of sediment from the interior of the houses; the mesh size is undefined (Cucchi 2005, 136).

*Çatalhöyük* is a huge tell settlement in the Konya Plain in Anatolia dated between the Pre-Pottery Neolithic, Ceramic Neolithic and Chalcolithic periods. The settlement consists of two mounds, the east one being larger than the west one. The occupation of the east mound has been dated between c.7.100 and c. 5.950 cal BC (Bayliss et al. 2015; Marciniak et al. 2015). The inhabitants constructed rectilinear mudbrick dwellings, attached to each other, with an entrance opening onto the roof; each household possessed ovens, storage bins and raised platforms, whereas open spaces are characterized by middens of household waste (Cessford 2001). The spiritual world of this society is reflected by a rich artistic repertoire: wall paintings, reliefs, figurines, sculptures incorporating bull horns; another spiritual aspect should be read into the numerous burials found below the house floors. The economy in the PPN levels was based on the cultivation of cereals and pulses as well as animal husbandry, mainly of sheep/goats; hunted game (wild ass, wild cattle, cervids and wild boar) added to the diet (Russell and Martin 2005; Fairbairn et al. 2005). Evidence for domestic cattle husbandry comes from the second half of the occupation. The *Mus* molars included in the GMM analysis were retrieved via extensive water flotation in 0.5 and 0.34 mm meshes of soil sediments, which originated from all PPN to Late Neolithic layers of the east mound and different contexts categories dated between 9.403-9.031 and 8.987-8.630 cal BP (Cessford 2005; Jenkins 2009, 68-70, 95-96; Bayliss et al. 2015); however, the ones targeted for aDNA analysis and ^14^C dating originate only from the PPN layers.

*Çafer Höyük* is an early to middle PPNB tell settlement in the upper Euphrates valley, typical of the so-called “Taurus PPNB”, which reflects an agglomeration of local Anatolian characteristics and elements from the middle Euphrates valley PPNB culture (Cauvin 1989). The layers XI and XII of the site have been ^14^C dated between 11.328-10.281 and 10.243-9.780 cal BP (Cauvin 1989). The architectural remains include rectangular buildings made of mudbrick. The inhabitants cultivated wild and domestic emmer and einkorn, wild peas and lentils, wild almonds and pistachios (Willcox 1998). They probably herded ‘agriomorphic’ livestock, meaning domestic animals with a wild morphology (referring specifically to sheep, goats and cattle), and simultaneously hunted ibexes and wild sheep available in the neighboring mountains and valley; thus the percentage between herded and hunted animals is not yet clear (Helmer 2008). The *Mus* molars included in this study originate from the middle PPNB layer which was water sieved using an undefined mesh size (Cucchi 2005, 136).

*Kissonerga-Mylouthkia* is a PPNB site near the southwest coast of Cyprus; no settlement or dwellings have been unearthed, with the exception of a pit and the foundations of a single building. The site comprises only seven wells, which were used for waste disposal after their drying (Peltenburg et al. 2001). Only three of these wells were dated: the filling of well Nr 116 dates between 10.684-10.296 and 10.491-10.178 cal BP (Peltenburg 2003); the filling of well Nr 133 dates between 9.294-9.009 and 9.080-8.645 cal BP (Peltenburg 2003); the filling of well 2030 dates at the Chalcolithic period (Cucchi 2005, 139). For all three wells, water sieving using a 1 mm mesh was effectuated for the recovery of small bones: Nr 116 (27 *Mus* molars used for GMM), Nr 133 (11 *Mus* molars used for GMM), Nr 2030 (59 *Mus* molars used for GMM) (Cucchi 2005, 138). Among these molars, only 5 specimens from well Nr 116 were used for aDNA analysis and AMS dating.

*Khirokitia* is a PPNC settlement in southeast Cyprus dated between 9.122-8.449 and 7.434-6.744 cal BP, which gave its name to the homonymous Aceramic Neolithic culture of the island, before the discovery of the PPN sites (Klimonas, Parekklisha-Skillourokambos, Kissonerga-Mylouthkia) (Dikaios 1953; Le Brun 1989 and 1994). The Khirokitia culture preserves some ‘old-fashioned’ elements, like the circular houses, in contrast to the rectangular ones of the PPNB/C mainland settlements; cattle are absent, despite their domestic/protodomestic status in contemporaneous sites of the mainland, and the villagers exploited sheep and goats (Vigne et al. 2000). In terms of architectural organization, these old-fashioned rounded buildings are grouped in clusters around open spaces, reminiscent of the rectangular multi-celled ones from contemporaneous continental sites (Le Brun 1989). The villagers cultivated cereals and legumes (hulled wheat, einkorn, barley, lentils, peas, bitter vetch, grass peas, fava beans and flax) and also collected wild fruit (almonds, pistachios, figs, acorns, olives) (Thiébault 2003; Parés and Tengberg 2017). A unique characteristic of Khirokitia is the exploitation of stone vases in the absence of pottery. Extensive water sieving of soil from the interior of the Khirokitia houses provided the 11 *Mus* molars included in this study (Cucchi 2005, 139).

*Cape Andreas-Kastros* is a PPNC settlement on the northeastern tip of the Karpasia Peninsula in Cyprus. Contemporaneous with the PPNC phase of Khirokitia, it belongs to the Khirokitia culture and is dated between 8.981-8.382 and 8.506-8.008 cal BP (Le Brun 1981; Le Brun and Daune-Le Brun 2003; Clarke et al. 2007). Basic differences between the two settlements are: 1) the lack of communal buildings in Cape Andreas-Kastros, and 2) the focus of its inhabitants on fishing and collecting shells from the nearby coast (Desse and Desse-Berset 2003). The 10 *Mus* molars included in this study were collected after water sieving of soils with an undefined mesh (Cucchi 2005, 139).

**The Ceramic Neolithic-Chalcolithic sites**

*Tol-e Nurabad* is a mound site, which was first occupied in the Neolithic period (c. 6.300-5.000/4.800 BC), with subsequent occupation during the Chalcolithic period (5^th^ millennium BC); occupation continued during the Bronze Age (4^th^ and 3^rd^ millennia BC) until historic times (Weeks et al. 2009). Two main phases of excavations were carried out in 2003 and 2008–2009. The site is important for the investigation of social transitions that occurred with the shift from village life to the period characterized by the appearance of highland cities. The Neolithic architecture consists of rectilinear buildings made of mudbrick and pisé, occasionally coated by thick mud plaster; but no architectural remains dating to the latest Neolithic have yet been revealed, though pottery is abundant. The site was occupied during the Chalcolithic period, producing remains of architecture in the Early Bakun period, *in situ* hearths dating to the Middle Bakun period, and Late Bakun period deposits (Trenches C and A; Weeks et al. 2009). After a possible gap in occupation, the site was (re-)occupied in the Lapui period, and then throughout the Early, Middle and Late Banesh periods (Weeks et al. 2009). The Bronze Age layers have so far not provided substantial architecture, only some superimposed floor-like deposits, probably activity and habitation areas in the vicinity of (as yet) uncovered houses (Trench A; Weeks et al. 2009). Several mudbrick walls and a floor of the later phases dating to the 2^nd^ and 1^st^ millennia BC have been exposed, which were disturbed by pits and holes (Trench B; Weeks et al. 2009). In terms of subsistence, caprids (sheep/goat) predominate in all periods in equal ratio, followed by cattle; very few bones of boar/pig, gazelle, dog and fox have been found in the Neolithic layers (Mashkour 2009). The 5 *Mus* mandibles included in this study originate from the Early Pottery Neolithic (7.838-7.681cal BP), Late Pottery Neolithic/Early Chalcolithic transition and the Chalcolithic layers (Early and Late Bakun periods, 6.665-6.499 cal BP), and were collected by dry sieving in 3 mm mesh (Weeks et al. 2009, 67).

*Tappeh Zagheh* is a mound settlement dated to the Late Neolithic (7.162-6.798 and 6.887-6.504 cal BP), Chalcolithc (5.370–5.070 to 4.240–4.460 cal BC) and the Bronze Age (Mashkour et al. 1999; Mashkour 2002); the archaeological evidence depicts a gradual transformation of the economic, cultural and social life of the society from a simpler to a more sophisticated level, probably reflecting some form of social hierarchy: the domestic buildings become more complicated, and there is a decorated building called the “painted temple”, which was adorned with mountain goat skulls and horns (Negahban 1979; Mollasalehi et al. 2006). During the Neolithic, domestic mammals were exploited (sheep, goats, cattle), along with hunted game (wild sheep and goats, gazelles, wild horses, hemiones, wild boar, red deer) (Mashkour 2002). Areas in Zagheh appear to have been zoned, according to particular activities: domestic areas contained walls, floors, pits, ovens, material culture (stone and copper tools, pottery, spindle whorls) and animal bones; kilns betrayed areas associated with the production of figurines, pottery and lamps; textile production must have occurred in areas that produced large numbers of spindle whorls (Mashkour 2001, 373). The *Mus* tooth was collected during the excavation of 2006 (directed by H. Mollah Salehi and M. Mashkour) in the Central Trench, where all the sediment from the trench was sieved with water. The *Mus* tooth was collected in a very secure context (Layer 131, RN 261, depth -558 to -567 in 2005) and is considered to have come from a commensal mouse.

*Ovçular Tepesi* is a settlement in Nakhchivan (Azerbaijan), which was occupied during the Late Chalcolithic I (4.350–4.250 cal BC) and II (4.250–3.940 BC) and the Early Bronze Age (2.900–2.650 cal BC) (Marro et al. 2009). The site is located on the Caucasian crossroads between Anatolia, the Iranian Plains and Mesopotamia. During the Middle Chalcolithic, the inhabitants used single-roomed rectangular semi-subterranean houses surrounded by posts for tent support. During the Late Chalcolithic, the rectangular houses rose above ground and were built using a pisé or mudbrick technique on a stone foundation; floors were made of beaten earth; in two of them, some sort of working surfaces, called ‘dials’, made of circular shaped pisé were discovered; several pits were also associated with these houses. The Early Bronze Age buildings are completely different: they are rounded, of no more than 7 m in diameter, and are constructed partially in mudbrick and partially in stone. Metallurgical activity must have happened in the settlement, as three copper tools found in a burial jar betray (Marro et al. 2011). Exchange networks with eastern Anatolia and the Kura Basin beyond the Caucasus are reflected in the on-site pottery (Gülçur and Marro 2012). The Chalcolithic meat diet was based mostly on ovicaprids, followed by cattle, pigs and wild game in small percentages; freshwater fish in abundant quantities supplemented the meat protein; the plant diet included mostly cereals and pulses (Berthon et al. 2013). The *Mus* molars originated from three Late Chalcolithic refuse pits, the fill of which was dated between 6.296-6.025 and 4.360–4.070 cal BP; they have already been classified as *domesticus* in an older study (Marro et al. 2009; Cucchi et al. 2013). The sediment that filled the pits was floated and sorted in a 1 mm mesh (Berthon et al. 2013).

*Norsun Tepe* is a tell site in the upper Euphrates valley dated to the Late Chalcolithic (5.991-5.749 cal BP), Early Bronze Age I (3310–2504 cal BC), Early Bronze Age II (2881–2614 cal BC), Early Bronze Age III (4.521-4.256 and 4.217-3.887 cal BP) and also the Iron Age (di Nocera 2000). Elaborate residential buildings with extensive storage features dated to the Bronze Age have been excavated. Metal mining (copper, arsenic, antimony) and subsequent metallurgic activity was taking place on site, reflected in relevant finds (smelting by-products, furnaces, moulds and artifacts). Expansive storing rooms with large vessels were uncovered inside the Early Bronze Age multi-roomed buildings (Hauptmann 2000). There is no available information for the sieving method and the mesh size for the retrieval of the *Mus* molars used in this study, which had primarily been published as *Mus musculus* ssp. by Kock et al. (1972).

*Chishko* is a site in the Adyghea Republic (North Caucasus) and belongs to the Maïkop culture of the 4^th^ to 3^rd^ millennium BC, which is known for the *kourgans*, the funerary mounds of the Chalcolithic period, and its wealth of metal objects (tools, weapons, vessels, ornaments). The site was initially excavated by Soviet archaeologists and then re-excavated by B. Lyonnet in 2000 and 2002. The Maïkop culture is thought to have been initially influenced by the Carpatho-Balkan metallurgical tradition and later by some northern Mesopotamian human incursion into the Georgian highlands, long before the arrival of southern Mesopotamian populations in the Upper Euphrates region (Lyonnet 2007, p. 17 and p. 150). The Chishko houses were constructed of wattle and daub, a tradition that still survives in the northwest Caucasus, and it is similar to sites from the earlier Tripol’ye culture, which extended from the Carpathian Mountains to the regions of Dnieper and Dniester (Riond in Lyonnet 2007). The houses were dated between 5.744-5.606 and 5.761-5.583 cal BP (Cabane 7) and 5.301-4.857 and 5.307-4.960 cal BP (Cabane 3) (Lyonnet 2007). The economy of many of the Maïkop sites, including Chishko, relied on animal husbandry and metal working rather than cereal agriculture (Hamon in Lyonnet 2007). The faunal remains of the site were studied by M. Mashkour in 2003 (unpublished report). The macro-mammalian remains are composed of mostly domesticates and a few wild species, including roe deer. Many microvertebrate remains were collected by the archaeologists in the settlement area through wet sieving soils from inside the huts; these are currently under study.

*Bucşani La Pod* is a tell settlement in southeast Romania, which was occupied during five occupation episodes separated by abandonment phases (Bem 2001). The uppermost occupation level dates to the Chalcolithic period and belongs to the Kodjadermen-Gumelniţa-Karanovo VI Balkan culture (4.500–3.900 BC). Seven burnt houses of this phase were excavated in the centre of the tell, considered as the result of intentional purifying fire, which aimed to finalize the life cycle of a dwelling (Chapman 1999). The *Mus* remains included in this study were found on the floor of the storage room of one of these burnt Chalcolithic houses after extensive wet sieving using a 1 mm mesh and were carbonized or calcined. This floor was ^14^C dated at 5.601-5.332 cal BP (Cucchi et al. 2011).

*Vinča-Belo Brdo* is a tell site in Serbia, which gave its name to the homonymous Late Neolithic Balkan culture of the late 6^th^ and early 5^th^ millennium. The life of the tell started before the Late Neolithic, during the Starčevo cultural phase, and was succeeded by the Vinča phase and later by Copper Age and Middle Bronze Age layers. The top of the tell was covered by an extended Medieval necropolis. The specimens included here derive from flotation samples taken during recent excavations of the uppermost Vinča period levels, led by Nenad Tasić of the University of Belgrade. The excavations revealed rows of closely spaced daub houses, destroyed in two successive fire events in the late 46^th^ century cal BC. House burnings are typical for late Vinča settlements, and the level of intention or symbolism behind them is widely debated (Tringham 1991 and 2005; Chapman 1999; Stevanović 2002). Most of the houses are rectangular and three-roomed, with the exception of a square one, which might have been used for storage. Three of the specimens described here derive from the destruction of House 01/06 during the earlier of these two fires, which has been ^14^C dated between 6510 and 6470 cal BP (Tasić et al. 2015). The fourth is from the burnt destruction layer of House 01/12.

*Mavropigi-Fillotsairi* is an Early (Ceramic) Neolithic settlement in northwest Greece providing evidence for the early agricultural communities that settled in this territory. It has been ^14^C dated between 8.305-8.034 and 8.036-7.935 cal BP (Karamitrou-Mentessidi et al. 2015). The inhabitants initially settled in two semi-subterranean structures, called pit-houses, with clay (earlier) or plaster (later) floors; the larger one possessed a round fire place and a main entrance. Through time the larger pit-house rose above the ground, reaching 100 m^2^ in size. During the latest habitation phase of the settlement, seven rectangular post-framed houses with clay floors were erected around the pit-houses. Some 100 pits, mostly for waste and rarely for storage, were found adjacent to the houses. Animals must have been kept in specific partitions in the village, as implied by various post holes. The pottery styles (polychrome and impresso ware) found in Mavropigi share affinities with central Greek Macedonia and Thessaly or with sites in the Korça basin (southwest Albania) (Bonga 2017). The villagers cultivated domestic cereals and pulses (emmer wheat, einkorn, barley, lentils) and collected wild fruits (pistachios, elderberries and cornelian cherries) (Valamoti 2011). Among the faunal remains, 88% belong to domestic animals (sheep, goats, pigs, cattle, dogs), whereas game has a small percentage (red and roe deer, aurochs, boars, foxes, hares and birds) (Michalopoulou 2017). The *Mus* molars included in this study were retrieved from the heavy residue after extensive water flotation of soil samples; the residue mesh had a 1 mm opening.

*Xirolimni-Portes* is an Early (Ceramic) Neolithic settlement in northwest Greece, close to Mavropigi-*Fillotsairi*, which has been ^14^C dated at 8.160-8.010 and 8.163-8.011 cal BP (Karamitrou-Mentessidi 2014). Habitation took place in clay-built dwellings founded on a stone structure, with clay floors and post-supported walls and roofs; the dwellings were destroyed by fire (Karamitrou-Mentessidi 2009). The archaeozoology of the site produced the same species as at *Mavropigi*-*Fillotsairi* (Michalopoulou 2017); the archaeobotanical remains point towards cultivation of domestic cereal and pulses (emmer, einkorn, barley, oats, lentils, peas or chickpeas) (as above). The *Mus* molars included in this study were retrieved from the heavy residue after extensive water flotation of soil samples in a residue mesh of a 1 mm opening.

*Dikili Tash* is a tell site in Eastern Macedonia (North Greece) dated between 6400 and 1100 BC, with diachronic occupation starting in the Early Neolithic and lasting until the Late Bronze Age, with an abandonment period during the Chalcolithic (4000 BC) (Malamidou et al. 2017; Lespez et al. 2013). The Early and Middle Neolithic phases are not yet excavated, but their presence has been revealed through coring in the tell, which has provided new ^14^C dates for the occupation sequence (Lespez et al. 2013). The life of the Late Neolithic settlement spans almost a millennium (6.719-6.297 to 5.282-4.887 cal BP); the houses were aligned along a NE-SW axis in parallel rows, separated by walkways. The rectangular houses were built of earth and wood, possibly following the wattle-and-daub technique; they were equipped with storage pits/bins occasionally coated with insulating clay material, waste pits, hearths and ovens with accompanying platforms for the food preparation (Malamidou et al. 2017). The diet of the villagers included cereals and pulses (einkorn, barley, lentils, peas, and bitter vetch), flax and fruits (acorns, wild pears, figs, blackberries, grapes); they made wine, probably the oldest attempt in Europe (Valamoti 2015). The *Mus* molars included in this study were retrieved from the heavy residue of flotated soil samples; the residue mesh had an opening of 1 mm. They dated mostly at the Late Neolithic habitation phase, although some of these contexts were mixed with later Early Bronze Age material.

*Avgi* is a Middle and Late Neolithic settlement in northwest Greece dated between 5.650-4.300 calBC (Stratouli et al. 2010, 2011 and 2014a). During the Middle and the early Late Neolithic, the villagers inhabited rectangular post-framed houses made of wattle and daub, and plastered with clay; large open areas for household activities separated the houses; the latter were destroyed by intense fire (Stratouli et al. 2010, 2011 and 2014a). These buildings were covered by extensive colluvial deposits, on top of which the inhabitants returned to continue their activities, as suggested by the pottery, the bioarchaeological and other finds and the ovens/hearths evidence, but they did not rebuild their houses. Towards the end of the site’s life, the villagers rebuilt some dwellings, from which we only recognize their foundation trenches and post-holes; several pits associated with these houses were also located (as above). The diet of the villagers included wild fruits (figs, cornelian cherries), cereals (barley, emmer, einkorn and lentils), freshwater fish and shellfish, mammal meat (sheep, goats, cattle, pigs, deer and wild boar). The *Mus* molars included in this study originate from both the Middle and Late Neolithic layers (7.409-7.172 and 7.307-7.167 cal BP, Stratouli 2013 in Greek) and were sorted from the heavy residue of water floated soil samples in 1 mm mesh.

*Theopetra* is a cave in Thessaly, Central Greece, whose stratigraphy spans the Paleolithic, Mesolithic and Neolithic periods (Kyparissi-Apostolika 2000, different chapters). It is thus very important in terms of diachronic paleoenvironmental reconstruction proxies, as well as for the documentation of early agricultural practices in Greece and the transition from harvesting to farming. The cave was diachronically used as both habitation and burial grounds; however, several geological factors dated after the Mesolithic period (karstic water activity, diagenesis) have obscured the distinction between the ceramic and preceramic Neolithic sub-layers. The Mesolithic groups that inhabited the cave collected wild fruits and nuts, wild pulses (lentils, vetch, vetchling, peas, and chickpea) and potentially harvested wild einkorn, namely the subspecies which is native to continental Greece; the Neolithic plant diet included the domestic forms of all these pulses, domestic cereals (einkorn, emmer, bread wheat and two species of barley), fruits and nuts; the animal kingdom is represented by the major domestic species and some hunted ones (mainly deer, boar, hare and badger) (Kyparissi-Apostolika and Kotzamani 2005; Hamilakis 2000). The *Mus* molars included in this study originate from the Neolithic layers, which were dated between 6.944-6.797 and 6.474-6.001 cal BP (Fakorellis et al. 2001). The *Mus* molars were retrieved after extensive dry sieving and water flotation of soil samples in 0.3 and 1 mm mesh (Kyparissi-Apostolika and Kotzamani 2005).

*Koutroulou Magoula* is a tell site on the border of Northern Phthiotida and Thessaly, Central Greece, dating to the transition from the Early to the Middle Neolithic and the Middle Neolithic itself (8.004-7.840 to 7.963-7.763 cal BP) (Hamilakis et al. 2017). The settlement was surrounded by ditches; other ditches within the inhabited part of the settlement, terracing, and a possible retaining wall were also attested through geophysical survey; the rectangular buildings were erected using mudbricks on stone foundations and possessed stone or mud floors with stone underfloor deposits; as some stone wall “foundations” survive to more than 1 m in height, there is a possibility that, in some buildings, walls were entirely made of stone. Open spaces between buildings, including a paved courtyard, staged daily activities, food preparation in hearths and probably feasts. The villagers herded sheep and goats, cattle, pigs and kept dogs; roe and red deer were hunted, and freshwater mussels were dug out of the local river bottoms; a combined soil-micromorphological, archaeobotanical and zooarchaeological study has shown that animal dung was abundantly present in all indoor and outdoor areas samples, indicating that herbivore animals were sharing the settlement space with humans, even the architecturally impressive buildings (Koromila et al. 2017a and 2017b). The cultivated plants include mainly emmer and einkorn—barley and oats are less common—followed by lentils, peas, grass peas and bitter vetch; figs, terebinth seeds, elder and wild plants were also used (as above). The *Mus* molars included in this study were retrieved in the heavy residue after extensive water flotation of soil samples using geological sieves with 0.3 and 1 mm meshes.

*Drakaina* is a cave site on the island of Kephallonia (western Greece), the stratigraphy of which spans from the Late Neolithic to the historic period (Stratouli et al. 1999 and 2014b). During the Late Neolithic (5.600-4.900/4.800 BC), Chalcolithic (4.900/4.800-3.700 BC) and Early Bronze Age (3.000-2.400 BC), the cave was periodically used by human groups for some sort of social/ritual activities, like feasting (Sratouli et al. 1999 and 2014b). These visits have been documented by the construction of several lime plastered floors, hearths or raked-out fire installations inside the cave; during these episodes, the human groups practiced in situ preparation and consumption of food (fruits such as almonds, figs, and grapes; pulses such as lentils, peas, dwarf chickling, lupin and Spanish vetchling; cereals such as emmer, einkorn, bread-wheat, barley and oat used for bulgur; meat of sheep/goat, cattle, pig, deer, hare; fish and shellfish) as well as deliberate stone knapping and arrowhead construction (as above). During the historic times the cave was used as a shrine devoted to deities of nature (Nymphs and Pan). The *Mus* molars included in this study were retrieved from the heavy residue after extensive water flotation of soil samples in geological sieves of 0.25–0.5 and 1 mm meshes from the Late Neolithic and Chalcolithic layers dating between 7.556-7.276 and 5.465-4.876 cal BP.

*Sarakenos* is a cave in Boeotia, Central Greece, the stratigraphy of which spans from the Middle/Upper Paleolithic, through the Mesolithic and Neolithic to the Middle Bronze Age (2^nd^ millennium BC) (Sampson et al. 2009). Therefore, it bears very important information for societal transition as well as climatic fluctuations from the Paleolithic to the Mesolithic, as well as from the Mesolithic to the Neolithic period. The Middle/Upper Paleolithic transition is marked by some Levallois type lithics but it has not been ^14^C dated. The Mesolithic communities that used the cave must have been isolated in the region, since they produced their microliths of local stone and practiced fowling in the nearby rocky crevices. The Early Neolithic is dated at 6.690–6.035 cal BC, when domestic sheep and goats appear, together with some hunted rock birds, and when the microliths are replaced by macroblades. The Middle Neolithic (5.790–4.200 cal BC) groups continued to seasonally occupy the cave, probably in association with their herding activities. The Late Neolithic (5.200–4.960 to 3.697–3.650 cal BC) occupation is of a more permanent character: several floors of beaten earth which supported posts for some sort of interior partitions have been uncovered,; a few clay pits/bins were also associated with a Late Neolithic floor, containing fragmented pottery, animal bones and clay figurines. Life in the cave continued during the Early (2.400–2.210 cal BC) and Middle Bronze Age, when the cave was abandoned. The *Mus* molars included in this study originate from the Early Bronze Age layer (4.409-4.159 cal BP); information on the sieving method are currently unavailable.

*Alepotrypa* is a deep cave in southeast Peloponnese (South Greece), the occupation of which is dated between 6100 and 3800 cal BC; the main activity periods, however are the Late and Final Neolithic, dated at 6.179-5.745 and 5.315-5.052 cal BP (Papathanasiou et al. 2018). The different chambers of the caves experienced different uses: the two closest to the entrance were used for primary and secondary burials; they contained lithic tools, coarse storage pottery, hearths, pits, clay floors, and short-term fires, indicating short-term occupation. The deepest and least accessible chamber, closest to the final lake chamber, gave the impression of a ritual area: numerous fragmented decorated pottery, scattered human bones, ornaments, unused lithic tools and an excess of burnt animal dung (as above, different chapters). The cave use must have stopped abruptly around 3800 BC due to an earthquake, which sealed the entrance of the cave and preserved the archaeological record without admixture from later activity. The *Mus* molars included in this study originate from the Late and Final Neolithic layers and were sorted from the heavy residue after soil flotation in 0.3, 0.5 and 1 mm meshes (Papayianni and Cucchi 2018).

**The Bronze Age sites**

*Ulug* *Depe* is a proto-urban tell site in Turkmenistan, the stratigraphy of which spans from the Late Neolithic (6.200–5.000 BC), Middle-Late Chalcolithic (4.000–3.000 BC) to the Middle Iron Age (1.100–329 BC) (Lecomte et al. 2002; MAFTUR 2015). The position of the site is crucial regarding the cultural interactions between Central Asia, Mesopotamia, Elam and the Indus Valley. The Neolithic period is represented by pottery fragments and lithics mixed with material of Chalcolithic layers, indicating the earlier occupation of the tell. The remains of the Chalcolithic and the Bronze Age are both domestic and industrial (numerous spindle whorls indicate domestic woolen textile manufacture, kilns associated with pottery and clay figurine production in the Chalcolithic; metallurgic installations in the Bronze Age); the Early Bronze Age settlement was fortified by a mudbrick wall. There are also funerary remains: some 50 tombs dated to various periods, from the Bronze Age to the Islamic period. Parts of an Iron Age citadel were uncovered above the Bronze Age remains. The economy of all periods relied on animal husbandry, mainly of ovicaprids, followed by cattle; hunting gazelles added an important meat component to the diet (Mashkour 2013; L’Huillier and Mashkour 2017). The *Mus* molars included in this study were retrieved through systematic soil flotation in 0.5 mm mesh from the Iron Age citadel. The microvertebrate remains of the site have been studied under the supervision of J.-D. Vigne (Berthon et al. in prep).

*Köhne Pāsgāh Tepesi* is a tell site on the Araxes river valley in northwest Iran, which was continuously occupied from the Late Chalcolithic (3.955–3.787 cal BC) to the end of the Early Bronze Age (2.817–2.665 cal BC); after a long hiatus, the site was reoccupied during the Iron Age and Parthian period (Maziar 2010 and in press 2019). There are no architectural remains from the Late Chalcolithic period, only on the northeastern side of the excavated trench, part of a possible arranged stone feature was recognized. Three bell shaped pits belong to this phase. They contain mudbrick remains, carbonized seeds, a copper pin, part of crucible, a broken animal figurine, and a sedimentary deposit filled with pottery and bones. There was no standardization of the pits as none of them were the same size. Of interest is that all of these three pits are located in a small area of about 25 m^2^. On the other parts of the Tepe, at least the southern and northwestern part, such pits were also recognized, and it is clear they are not limited to just one part of the site.

The earliest occupation of the Early Bronze Age, which is represented by the Kura-Araxes material culture, is marked by 12 rows of refuse debris but no architecture, perhaps indicating a temporary or seasonal use of the site; in later phases of the Early Bronze Age, a circular building with mudbrick wall and a thatched roof was built; a series of floors, pits and two manqàls (hearths) are associated with this building. The faunal remains of this site were studied by F. A. Mohaseb and M. Mashkour (Mohaseb 2012, Davoudi et al. 2018). The microvertebrate remains were collected through flotation. The *Mus* molars included in this study are dated to the Early Bronze Age (4.767-4.615 cal BP) and originate from the refuse pits of trench B, contexts L6050 (Phase IV), 6065, 6070 (Phase IIIa) and 6071 (Phase II).

*Agia Triada* is a very deep karstic cave on the south tip of Euboea Island near continental Greece, the stratigraphy of which spans the Late (4.896/4.329–4.360/4.053 cal BC) and Final Neolithic (5.914-5.663 cal BP) and the Early Bronze Age I-II (4.815-4.527 cal BP) (Mavridis and Tankosić 2016 a&b). The shape of the cave and the archaeological finds suggest a ritual and burial use for the Early Bronze Age layers, whereas the Late/Final Neolithic use is still unclear. The Late Neolithic layers contained mixed animal and human bones, evidence for the existence of pits and hearths as well as a ‘feature’ made of hard burnt soil, potsherds and rock slabs, below which a prosopomorphic jar handle was found in an ash layer (Mavridis and Tankosic 2016a). The Final Neolithic use of the cave consisted mainly of a paved floor, onto which most of the material was found (pottery, animal bones, spindle whorls, ground and chipped stone tools) (Mavridis and Tankosic 2016a). Since this evidence comes from a dark and deep area of the cave, a ritual or symbolic use may be possible. After a significant time hiatus, the cave was used as a burial place during the middle Early Bronze Age; the corpses were laid on the floor of the cave, on top of a layer of burnt grain and grave offerings (personal items and pottery) (Mavridis and Tankosic 2016b). The single *Mus* molar included in this study is dated to the hiatus before the Early Bronze Age burials, in a layer potentially affected by karstic water activity during prehistory; it was retrieved after soil flotation in 0.3 and 1 mm meshes.

*Akrotiri* is a large Bronze Age town on Santorini Island in the Aegean; the earliest habitation is dated to the Early Bronze Age (4.438-4.257 cal BP), when the people used artificial caves dug into the natural soft bedrock of the island as dwellings. During the Middle (2.130–1.980 to 1.881–1.755 cal BC, 2σ) and the Late Bronze Age (3.557-3.380 cal BP) (Maniatis 2012), the habitation pattern changed: multi-storeyed buildings were erected along narrow streets, some of which were elegantly adorned with frescoes. The buildings comprised storage rooms with vessels, kitchens, private apartments and lounges for domestic activities such as textile manufacture or socializing; there was a sewage system running under the streets of the town. The economy of the settlement was a mixture of agricultural activities (wine and olive oil production, cereal and pulse cultivation), animal husbandry, fishing, metallurgy and commerce, since numerous artefacts of ‘exotic’ provenance, such as two ostrich eggs rhyta, as well as pottery from different origins have been found; all the evidence points to a proto-urbanization for the settlement (Doumas 2010 and 2013). Life in the town stopped suddenly due to a huge volcanic eruption dated to between 1622–1548 cal BC (Maniatis 2012). The *Mus* molars included in the GMM study span the Bronze Age sequence and were retrieved from the heavy residue after extensive soil flotation in 0.25, 0.5 and 1 mm meshes; the mandibles submitted to aDNA and ^14^C dating derive from the Early Bronze Age layers and the transition between the Early and the Middle Bronze Age.

*Mochlos* is a Bronze Age town built on a small islet a few metres off the northeast coast of Crete; the town was occupied during the Early (4.849-4.440 to 3.550-3.274 cal BP), Middle (4.515-3.722 cal BP) and Late Bronze Age (3.550-3.274 cal BP) (Switsur et al. 1970; Switsur and West 1972; Warren 1976; Burleigh et al. 1977; Manning 1995 and 2010; Manning et al. 2006); the last is the main occupation phase of the town (Soles and Davaras 1992). The inhabitants practiced agriculture and husbandry on the fertile opposite coastal valley; the naturally protected harbor served their commercial activity in both imports and exports of crafts, since a specialized Late Bronze Age artisan’s quarter has been excavated: evidence for pottery production, jewelry creation, stone-vase crafting (Soles and Davaras 2003). The houses comprised living and storing quarters; a ritual/ceremonial building with 3 floors has also been found. The *Mus* molars included in the GMM study span the Bronze Age sequence and were retrieved from the heavy residues of extensively floated soil samples in 0.3, 0.5 and 1 mm meshes (Papayianni 2012); the mandibles submitted to aDNA and ^14^C dating derive from the Early and Late Bronze Age layers.

*Malia* is a Bronze Age town on the north coast of Crete, dated to and built around a Middle-Late Bronze Age palace, i.e. a large building complex including living quarters, large storage rooms, shrines, open areas and workshops. The town that evolved around the palace consisted of quarters, one of which (Mu) was an artisan’s quarter for the production of seals, stone vases and pottery. Malia was a significant port, bearing well evidenced relationships with Anatolia, Near East and especially Egypt (Poursat 2010). The *Mus* molars included in this study originate from Quarter Pi and were retrieved through systematic soil flotation of all layers in 0.25, 0.5 and 1 mm meshes. Quarter Pi lies west of the palace and exhibits a long urban history from the Early (4.849-4.440 to 3.550-3.274 cal BP) to the Late Bronze Age (3.550-3.274 cal BP). A large Late Bronze Age building (3.550-3.274 cal BP) which was divided into many rooms of different sizes and usage, either domestic or industrial as well as ritual, according to the variety of the finds, was discovered. Below and around it there is evidence for Middle Bronze Age buildings (5.435-3.716 cal BP) (Delibrias et al. 1970; Warren 1976; Burleigh et al. 1977; Manning 1995 and 2010; Manning et al. 2006; Gomrée et al. 2012). The study of the stratigraphy is still in progress.

*Chania-GSE* are two different excavating campaigns in the Bronze Age town of Chania, below the Kastelli hill, in the heart of the modern city of West Crete, which have uncovered different parts of the prehistoric occupation. The earliest habitation occurred in the Final Neolithic/Early Bronze Age transition (ca. 3.500 BC); a series of deep narrow pits dug in the natural bedrock have been unearthed on top of the Kastelli hill of the modern city, which must have functioned as postholes to support houses (Andreadaki-Vlazaki 2010). During the Early Bronze Age (3.000–1.900 BC) there must have been a settlement on Kastelli hill, evidenced by the discovery of plastered walls, clay floors, hearths, as well as local and imported pottery. During the Middle Bronze Age (1.900–1.700 BC) habitation on the Kastelli hill continues but the available evidence is scarce, due to the superimposition of layers of later periods; nevertheless, they suggest a gradual reinforcement of the marine commercial activities (Andreadaki-Vlasaki 2010). The Late Bronze Age (1.700–1.250 BC) is the main occupation phase, during which there must have been a palace in the town, since Linear A and B tablets have been found, which mention the name of the town: *ku-do-ni-ja*. The private houses had two storeys and were decorated with frescoes; they had private, public and storage rooms; open areas for domestic or industrial activities separated them. Apart from the houses, a building complex of different rooms of some ritual or symbolic function has come to light, which is considered to be the shrine of the nearby still elusive palace (Andreadaki-Vlasaki 1999; Hallager and Hallager 2003). The *Mus* molars included in this study originate from all the layers of both campaigns and were sorted from the heavy residues after systematic soil flotation in 0.25, 0.5 and 1 mm meshes (Papayianni 2012). The mandibles subjected to aDNA analysis and ^14^C dating originate from the Early and the Late Bronze Age layers (3.835-3.450 and 3.558-3.181 cal BP) (Housley et al. 1999).

*Uluburun shipwreck* is dated to the Late Bronze Age and is located off the southwest Turkish coast near the modern city of Kaş, Antalya province (Pulak 1996). The vessel had a capacity of 20 tons and was carrying a variety of items, including Mycenaean (Late Bronze Age-Late Helladic IIIA:2) pottery, which postdates all the aforementioned Bronze Age sites; the dendrochronology of wood found on the vessel gave an absolute date of 3.338-3.336 cal BP or 1.316–1.305 BC (Kuniholm et al. 1996). A total of 10 tons of copper and tin ingots was its main cargo; exotic items, such as ostrich eggs, ebony, amber, elephant and hippopotamus ivory, Cypriot pottery, Canaanite storage jars are just some of the evidence for a multi-stop voyage that connected different ports in different regions. The vessels are thought to have been part of a royal command, due to the wealth of its cargo (Pulak 1998). A mouse mandible was found among the sieved material from the excavation, which has already been attributed to a *domesticus* of Syrian origin, adding to the evidence for the ship’s route and its last port embarkation before sinking (Cucchi 2008).

**REFERENCES**

Andreadaki-Vlazaki, M. 1999. Minoan Kydonia. In Tzedakis, Y., Martlew, H. (eds.), *Minoans and Mycenaeans: flavours of their time*, Greek Ministry of Culture, Athens, 104-105.

Andreadaki-Vlazaki, M. 2010. *Chania (Kydonia): a tour in places of ancient memory*. Greek Ministry of Culture, 25^th^ Ephorate of Prehistoric and Classical Antiquities, Chania (in Greek).

Arbez, L., 2018 Faune et environnement passés du Sud de la Mer Caspienne : Analyse paléoécologique et taphonomique des restes de micromammifères du site d’Ali-Tappeh. Master Thesis, Quaternaire & Prehistoire, Muséum national d’Histoire naturelle, Paris. 110 p.

Bar-Yosef, O., A. Gopher, E. Tchernov and M. Kislev. 1991. Netiv Hagdud: an Early Neolithic village site in the Jordan Valley. *J. Field Archaeol.* 18 (4), 405-426.

Bayliss, A., Brock, F., Farid, S., Hodder, I., Southon, J., Taylor, R.E. 2015. Getting to the bottom of it all: a Bayesian approach to dating the start of Çatalhöyük, Journald of World Prehistory 28, 1-26. DOI 10.1007/s10963-015-9083-7

Bem, C. 2001. Noi propuneri pentru o schiţă cronologică a eneoliticului româ­nesc. *Pontica* 33–34, 25–121.

Berthon R, Decaix A, Kovács ZE, Van Neer W, Tengberg M, Willcox G, Cucchi T. 2013. A bioarchaeological investigation of three chalcolithic pits at Ovçular Tepesi (Nakhchivan, Azerbaijan). *Journal of* *Environmental Archaeology* 18 (3), 191-200.

Biglari, F., M. Javeri, M. Mashkour, Y. Yazdi, S. Shidrang, M. Tengberg, and K. Taheri. 2009. Test excavations at the Middle Paleolithic sites of Qaleh Bozi, Southwest of Central Iran, A preliminary report. In M. Otte, F. Biglari, and J. Jaubert (eds.), *Iran Palaeolithic*. Proceedings of the XV World Congress UISPP, Lisbon, Vol. 28, BAR International Series 1968, 29-38.

Biglari F, M. Mashkour, S. Shidrang, M. Javeri, Y. Yazdi, M. Tengberg, Bahrololumi, F. Darvish, J and K. Taheri, 2015. Qaleh Bozi, New Evidence of Late Middle Paleolithic Occupation in the Zayandeh-Rud Basin, Esfahan Province, *Archaeological Research of Iran,* Vol. 4 No. 7, 7-26

Bonga L.A. 2017. Thoughts on the preliminary study of Early Neolithic decorated pottery from the central origma at Mavropigi-Filotsairi. In Sarris, A., Kalogiropoulou, E., Kalayci, T., Karimali, L. (eds.), *Communities, landscapes, and interaction in neolithic Greece: proceedings of the international conference, Rethymno 29-30 May, 2015*. International Monographs in Prehistory, Archaeological Series 20, Michigan, USA, 374-387.

Bridault, A., Rabinovich, R., Simmons, T. 2008. Human activities, site location and taphonomic process: A relevant combination for understanding the fauna of Eynan (Ain Mallaha), level IB (Final Natufian), Israel. In E. Vila, L. Gourichon, A. Choyke & H. Buitenhuis (eds.), *Archaeozoology of the Near East VIII*,. Lyon: Maison de l’Orient méditerranéen, pp. 99–117.

Burleigh, R., Hewson, A., and Meeks, N. 1977. British Museum Natural Radiocarbon Measurements IX, *Radiocarbon* 19 (2), 143-160.

Cauvin, J. 1989. La Stratigraphie de Çafer Höyük-Est (Turquie) et les origines du PPNB du Taurus. *Paléorient* 15 (1), 75-86.

Cessford, C. 2001. A new dating sequence for Çatalhöyük, *Antiquity* 75, 717-725.

Cessford, C. 2005. Absolute dating at Çatalhöyük, in Hodder, I. (ed.) *Changing materialities at Çatalhöyük: reports from the 1995–1999 seasons*, Cambridge: McDonald Institute, 65-100.

Chapman, J. 1999. Burning the ancestors: deliberate housefiring in Balkan prehistory. In A. Gustafsson & H. Karlsson (eds.) *Glyfer och arkeologiska rum—en vanbok till Jarl Nordbladh*. Gothenburg: Institute of Archaeology, 113-126.

Clarke J., McCartney C., Wasse A. 2007. *On the Margins of Southwest Asia. Cyprus during the 6th to 4th Millennia BC*. Oxford, Oxbow Books.

Coquegniot, E. 1998. Dja'de el Mughara (moyen-Euphrate), un village néolithique dans son environnement naturel à la veille de la domestication. In M. Fortin and O. Aurenche (eds.), *Espace naturel, espace habité en Syrie du Nord (10e - 2e millénaires av. J.-C.) / Natural Space, Inhabited Space in Northern Syria (10th - 2nd millennium B.C.)*. Actes du colloque tenu à l'Université Laval (Québec) du 5 au 7 mai 1997. Lyon : Maison de l'Orient et de la Méditerranée Jean Pouilloux, 1998. Travaux de la Maison de l'Orient méditerranéen, 28, 109-114.

Cucchi T. 2005. Le commensalisme murin et les premières sociétés méditerranéennes. Unpublished PhD Thesis, Muséum National d’Histoire Naturelle, Paris.

Cucchi, T. 2008. Uluburun shipwreck stowaway house mouse: molar shape analysis and indirect clues for the vessel's last journey, *Journal of Archaeological Science* 35, 2953-2959.

Cucchi, T., Bălăşescu, A., Bem, C., Radu, V., Vigne, J.-D. and Tresset, A. 2011. New insights into the invasive process of the eastern house mouse (*Mus musculus musculus*): Evidence from the burnt houses of Chalcolithic Romania. *The Holocene* 21 (8), 1195-1202.

Cucchi, T, Kováks, Z.-E., Berthon, R., Orth, A., Bonhomme, F., Evin, A., Siahsarvie, R., Darvish, J., Bakhshaliyev, V. and Marro, C. 2013. On the trail of Neolithic mice and men towards Transcaucasia: zooarchaeological clues from Nakhchivan (Azerbaijan), *Biological Journal of the Linnean Society* 2013, 1-12.

Davoudi H., Berthon R., Mohaseb A., Shiva Sheikhi S., Abedi A., Mashkour M, 2018. Kura-Araxes Exploitation of Animal Resources in Northwestern Iran and Nakhchivan. In Çakirlar C., Chahoud J., Berthon R., Pilaar Birch S. (eds.), *Archaeozoology of the Near East 12. Proceedings of the Groningen 2015 ASWA conference. Groningen Archaeological Series*, Groningen, 91-108.

Delibrias, G., Guillier M.T. and Labeyrie, J. 1970. Gif Natural Radiocarbon Measurements V, *Radiocarbon* 12, 421-443.

Desse, J., Desse-Berset, N. 2003. Les premiers pêcheurs de Chypre. In J. Guilaine et A. Le Brun (eds.), *Le Néolithique de Chypre*, vol. Supplément 43. Paris : Bulletin de Correspondance Hellénique, 279-291.

Dikaios, P. 1953. *Khirokitia*. Oxford University Press.

di Nocera, G.-M. 2000. Radiocarbon datings from Arslantepe and Norşuntepe: The fourth- third millennium absolute chronology in the Upper Euphrates and Transcaucasian region. In: *Chronologies des pays du Caucase et de l’Euphrate aux IVe-IIIe millénaires. From the Euphrates to the Caucasus: Chronologies for the 4th-3rd millennium B.C. Vom Euphrat in den Kaukasus: Vergleichende Chronologie des 4. und 3. Jahrtausends v. Chr. Actes du Colloque d’Istanbul, 16-19 décembre 1998*. Istanbul : Institut Français d'Études Anatoliennes-Georges Dumézil, *Varia Anatolica* 11, 73-93.

Doumas, C.G., 2010. Akrotiri. In Cline, E.H. (ed.), *The Oxford Handbook of the Bronze Age Aegean (ca. 3000–1000* BC*)*, Oxford, 752–61.

Doumas, C.G. 2013. Akrotiri, Thera: reflections from the East. In Aruz, J., Graff, S.B., Rakic, Y. (eds.), *Cultures in contact: from Mesopotamia to the Mediterranean in the 2^nd^ Millennium BC*. Yale University Press, New Haven and London, 180-187.

Fakorellis, Y., N. Kyparissi-Apostolika, and Y. Maniatis. 2001. The cave of Theopetra, Kalambaka: Radiocarbon evidence for 50,000 years of human presence. *Radiocarbon* 43(2), 975-994.

Fairbairn, A., Near, J., Martinoli, D. 2005. Macrobotanical investigation of the North, South and KOPAL Area Excavations at Çatalhöyük East. In Hodder, I. (ed.), *Inhabiting Çatalhöyük: reports from 1995-99 seasons.* Çatalhöyük Project Volume 4, McDonald Institute Monographs/BIAA 38, Cambridge and London, 137-202.

Gomrée, T., Langohr, C., Pomadère, M. 2012. Excavations in the Pi area at Malia (2005-2010). In Andrianakis, M., Varthalitou, P. and Tzachili, I. (eds.), *Archaeological work in Crete 2, Proceedings of the 2^nd^ Meeting, Rethymnon 26-28 November 2010*, Faculty of Letters Publications, University of Crete, 89-97.

Gülçur, S. and Marro, C. 2012. The view from the North: comparative analysis of the Chalcolithic pottery assemblages from Norşuntepe and Ovçular Tepesi. In Marro, C. (ed.), *After the Ubaid: interpreting change from the Caucasus to Mesopotamia at the dawn of urban civilization (4500–3500 BC), Varia Anatolica 27*. Istanbul: Institut Français d’Études Anatoliennes Georges – Dumézil, 305–352.

Hallager, E. and Hallager, B. P. 2003. *The Greek-Swedish Excavations at the Agia-Aikaterini Square Kastelli, Khania 1970-1987 and 2001, Vol.III1: The Late Minoan IIIB:2 Settlement*, Acta Instituti Atehniensis Regni Sueciae, Series in 4o, XLVII:III:1, Stockholm.

Hamilakis, Y. 2000. Zooarchaeology of Neolithic Theopetra. In Kyparissi-Apostolika, N. (ed) *Theopetra: Ten Years of Research*, Athens: Ministry of Culture (in Greek), 263-66..

Hamilakis, Y., Kyparissi-Apostolika, N., Loughlin, T., Carter, T., Cole, J., Facorellis, Y., Katsarou, S., Kaznesi, A., Pentedeka, A., Tsamis, V., Zorzin, N. 2017. Koutroulou Magoula in Phthiotida, Central Greece: a Middle Neolithic Tell site in Context. In Sarris, A., Kalogiropoulou, E., Kalayci, T., Karimali, L. (eds.), *Communities, landscapes, and interaction in neolithic Greece: proceedings of the international conference, Rethymno 29-30 May, 2015*. International Monographs in Prehistory, Archaeological Series 20, Michigan, USA, 81-96.

Hauptmann, H. 2000. Zur Chronologie des 3. Jahrtausends v. Chr. am oberen Euphrat Aufgrund der Stratigraphie des Norşuntepe. In: *Chronologies des pays du Caucase et de l’Euphrate aux IVe-IIIe millénaires. From the Euphrates to the Caucasus: Chronologies for the 4th-3rd millennium B.C. Vom Euphrat in den Kaukasus: Vergleichende Chronologie des 4. Und 3. Jahrtausends v. Chr. Actes du Colloque d’Istanbul, 16-19 décembre 1998*. Istanbul : Institut Français d'Études Anatoliennes-Georges Dumézil, *Varia Anatolica* 11, 419-438.

Helmer, D. 2008. Révision de la faune de Çafer Höyük (Malatya, Turquie) : apports des méthodes de l’analyse des mélanges et de l’analyse de Kernel à la mise en évidence de la domestication. In J. Pouilloux (ed.), *Archaeozoology of the Near East VIII. Actes des huitièmes Rencontres internationales d'Archéozoologie de l'Asie du Sud-Ouest et des régions adjacentes*. Lyon : Maison de l'Orient et de la Méditerranée vol.49, 169-195.

Helmer, D., Gourichon, L., Monchot, H., Peters, J., Saña Segui, M. 2005. Identifying early domestic cattle from Pre-Pottery Neolithic sites on the Middle Euphrates using sexual dimorphism. In J.-D. Vigne, J. Peters and D. Helmer (eds.), *First steps of animal domestication: new archaeozoological approaches.* Proceedings of the 9th Conference of the International Council of Archaeozoology (Durham, 23rd-28th August 2002), Oxford: Oxbow books, 55-60.

Hole, F., Flannery, K.V., Neely, J.A. 1969. *Prehistory and human ecology on the Deh Luran plain*. Memoirs of the Museum of Anthropology, no. 1. Ann Arbor: University of Michigan Press.

Housley, R.A., Manning, S.W., Cadogan, G., Jones, E.R., Hedges, R.E.M., 1999. Radiocarbon, calibration and the chronology of the Late Minoan IB Phase. *Journal of Archaeological Science* 26, 159-171.

Ibáñez, J. J. 2008a. Introduction, in Ibáñez, J. J. (ed.), *Le site néolithique de Tell Mureybet (Syrie du Nord). En hommage à Jacques Cauvin*, BAR International Series 1843, Oxford: [Archaeopress](https://en.wikipedia.org/wiki/Archaeopress), 7–13.

Ibáñez, J. J. 2008b. Conclusion, in Ibáñez, J. J. (ed.), *Le site néolithique de Tell Mureybet (Syrie du Nord). En hommage à Jacques Cauvin*, BAR International Series 1843, Oxford: Archaeopress, 661–675.

Jaubert, J., Biglari, F., Crassard, R., Mashkour, M., Rendu, W., Shidrang, S. 2010. Paléolithique moyen récent de la grotte de Qaleh Bozi 2 (Ispahan, Iran): premiers résultats de la campagne 2008. *Iranian Archaeology* 1, 21-32.

Jenkins, E. 2009. Unwanted inhabitants? The Microfauna from Çatalhöyük and Pinarbasi, Cambridge.

Karamitrou-Mentessidi, G. 2009. Aiani and Kozani prefecture: ten years of research (in Greek). In P. Adam-Veleni and K. Tzanavari (eds.), *20 years of* *Archaeological Excavations at Macedonia and Thrace*, Greek Ministry of Culture and Aristoteleian Univeristy of Thessaloniki, Thessaloniki, Greece, 105-126.

Karamitrou-Mentessidi, G. 2014. About prehistoric Sites in west Macedonia: prefectures of Kozani and Grevena (in Greek), in Stefani, E., Merousis, N. Dimoula, A. (eds.), *A century of research in Prehistoric Macedonia: 1912-2012, International Conference Proceedings, Archaeological Museum of Thessaloniki 22-24/11/2012*, Thessaloniki, 233-250.

Karamitrou-Mentessidi, G., Efstratiou, N., Kaczanowska, M., Kozłowski, J.K. 2015. Early Neolithic Settlement of Mavropigi in western Greek Macedonia, *European Prehistory* 12 (1-2), 47-116.

Kock, Von D., Malec, F., Storch, G. 1972. Rezente und subfossile Kleinsäuger aus dem Vilayet Elazig, Ostanatolien. *Z. Säugetierkunde* 37, 204-229.

Koromila, G., P. Karkanas, G. Kotzamani, K. Harris, [Y. Hamilakis](https://vivo.brown.edu/display/yhamilak), and N. Kyparissi-Apostolika. 2017a. Humans, Animals, and the Landscape in Neolithic Koutroulou Magoula, Central Greece: An Approach through Micromorphology and Plant Remains in Dung. In Sarris, A., Kalogiropoulou, E., Kalayci, T., Karimali, L. (eds.), *Communities, landscapes, and interaction in neolithic Greece: proceedings of the international conference, Rethymno 29-30 May, 2015*. International Monographs in Prehistory, Archaeological Series 20, Michigan, USA, 269-280.

Koromila, G., P. Karkanas, [Y. Hamilakis](https://vivo.brown.edu/display/yhamilak), N. Kyparissi-Apostolika, G. Kotzamani and K. Harris, 2017b. The Neolithic tell as a multi-species monument: Human, animal, and plant relationships through a micro-contextual study of animal dung remains at Koutroulou Magoula, central Greece. *Journal of Archaeological Science: Reports* 19, 753-768.

Kuniholm, P.I., Kromer, B., Manning, S.W., Maryanne, N., Latini, C.E., Bruce, M.J., 1996. Anatolian tree rings and the absolute chronology of the eastern Mediterranean, 2220–718 BC. *Nature* 381, 780–783.

Kyparissi-Apostolika, N. Kotzamani, G. 2005. Worlds in transition: Mesolithic/Neolithic lifestyles at the cave of Theopetra, Thessaly/Greece. In Lichter, G. (ed.), *How did farming reach Europe?* BYZAS 2, 173-182.

Kyparissi-Apostolika, N. 2000 (Ed.). *"Theopetra Cave - Twelve years of excavation and research 1987-1998"*; Proceedings of the International Conference, Trikala 6-7 November 1998, Greek Ministry of Culture.

Lecomte, O., Francfort, H.-P., Boucharlat, R. et Mamedow, M. 2002. Recherches archéologiques récentes à Ulug Dépé (Turkménistan), *Paléorient* 28 (2), 123-131.

Le Brun, A. 1981. *Un site néolithique précéramique en Chypre : Cap Andreas Kastros*, A.D.P.F. édition. Paris.

Le Brun, A. 1989. Fouilles récentes à Khirokitia (Chypre), 1983-1986, *Éditions Recherche sur les Civilisations*. Paris.

Le Brun, A. 1994. Fouilles récentes à Khirokitia (Chypre), 1988-1991, *Éditions Recherches sur les Civilisations*. Paris.

Le Brun, A., Daune-Le Brun, O. 2003. Deux aspects du Néolithique pré-céramique récent de Chypre: Khirokitia et Cap Andreas-Kastros. In J. Guilaine et A. Le Brun (eds.), *Le Néolithique de Chypre,* vol. Supplément 43, Bulletin de Correspondance Hellénique, 45-59.

Lespez, L., Tsirtsoni, Z., Darcque, P., Koukouli-Chrysanthaki, H., Malamidou, D., Treuil, R., Davidson, R., Kourtessi-Philippakis, G., Oberlin, C., 2013. The lowest levels at Dikili Tash, Northern Greece: a missing link in the Early Neolithic of Europe. *Antiquity* 87, 30–45.

L’Huillier J. and Mashkour M. 2017. Animal exploitation in the oases. An archaeozoological review of Iron Age sites in southern Central Asia (Uzbekistan and Turkmenistan). 2017. *Antiquity*. 91 (357), 655-673

Lyonnet B. 2007. La culture de Maikop, la Transcaucasie, l'Anatolie orientale et le Proche-Orient: relations et chronologie. In B. Lyonnet (ed.), *Les cultures du Caucase (VIe-IIIe millénaires avant notre ère). Leurs relations avec le Proche Orient*, Paris: CNRS Éditions, 133–161.

MAFTUR 2015. Mission Archéologique Franco-Turkmène Ministère des Affaires Étrangères – CNRS Scientific concept: Annie Caubet with contributions of G. Davtian, J.-F. Haquet, C. Hamon, J. Lhuillier, M. Mashkour and M. Tengberg.

Malamidou, D., Ntinou, M., Valamoti, S.-M., Tsirtsoni, Z., Koukouli-Chrysanthaki, H., Darcque, P. 2017. An Investigation of Neolithic Settlement Pattern and Plant Exploitation at Dikili Tash: Reconsidering Old and New Data from the late 5th Millennium B.C. Settlement. In Sarris, A., Kalogiropoulou, E., Kalayci, T., Karimali, L. (eds.), *Communities, landscapes, and interaction in neolithic Greece: proceedings of the international conference, Rethymno 29-30 May, 2015*. International Monographs in Prehistory, Archaeological Series 20, Michigan, USA, 60-80.

Manca L., M. Mashkour, Shidrang S., A. Averbouh, F. Biglari. 2018. Bone, shell tools and ornaments from the Epipalaeolithic site of Ali Tappeh, East of Alborz Range, Iran. *Journal of Archaeological Science: Reports* 21, 137-157. DOI: 10.1016/j.jasrep.2018.06.023

Maniatis, Y. 2012. Radiocarbon dating of the Late Cycladic building and destruction phases at Akrotiri, Thera: new evidence, *The* *European Physical Journal Plus* 127, 9.

Manning, S.W. 2010. Chronology and Terminology. In In Cline, E.H. (ed.), *The Oxford Handbook of the Bronze Age Aegean (ca. 3000–1000* BC*)*, Oxford, 91-152.

Manning, S. W. 1995. *The Absolute Chronology of the Aegean Early Bronze Age: Archaeology, History, and Radiocarbon*. Monographs in Mediterranean Archaeology 1. Sheffield: Sheffield Academic Press.

Manning, S.W, Ramsey, C.B., Kutschera, W., Higham, T., Kromer, B., Steier, P. Wild, E.M. 2006. Chronology for the Aegean Late Bronze Age 1700-1400 B.C. *Science* 312, May 2006. DOI: 10.1126/science.1125682

Marciniak, A., Barański, M.Z., Bayliss, A., Czerniak, L., Goslar, T., Southon J. and Taylor, R.E. 2015. Fragmenting times: interpreting a Bayesian chronology for the Late Neolithic occupation of Çatalhöyük East, Turkey. *Antiquity*, 89, 154-176 doi:10.15184/ aqy.2014.33

Marro, C., Bakhshaliyev, V., Ashurov, S. 2009. Excavations at Ovçular Tepesi (Nakhchivan, Azerbaijan). First preliminary report: the 2006-2008 seasons. *Anatolia Antiqua* 17, 31-87.

Marro, C., Bakhshaliyev, V., Ashurov, S. 2011. Excavations at Ovçular Tepesi (Nakhichevan, Azerbaijan). Second preliminary report: the 2009–2010 seasons. *Anatolia Antiqua* 19, 53–100.

Mashkour, M. 2001. *Chasse et élevage du Néolithique à l'Âge du Fer dans la plaine de Qazvin (Iran). Étude archéozoologique des sites de Zagheh, Qabrestan et Sagzabad.* Unpublished PhD thesis, Université de Paris I-Sorbonne.

Mashkour M. 2002. Chasse et élevage au Nord du Plateau central iranien entre le Néolithique et l’Âge du Fer. *Paléorient* 28(1), 27-42.

Mashkour M. 2003. Rapport préliminaire sur l’étude de la faune de Chishkho (République d’Adygei- Russie). French Ministry of Foreign Affairs. Unpublished Report.

Mashkour, M. 2009. Faunal remains from Tol-e Nurabad and Tol-e Spid. In D. T. Potts, K. Roustaei, C. A. Petrie and L. R. Weeks (eds.), *The Mamasani Archaeological Project Stage One: A Report on the First Two Seasons of the ICAR – University of Sydney Expedition to the Mamasani District, Fars Province, Iran*. BAR International Series 2044. Oxford, Archaeopress, 135-146.

Mashkour M. 2013. Sociétés pastorales et économies de subsistance au Nord Est de l’Iran et au Sud du Turkménistan. In Bendezu-Sarmiento, J. (dir.), Archéologie française en Asie centrale. Nouvelles recherches et enjeux socioculturels, *Cahiers d’Asie Centrale*, n° 21/22 , 533-544

Mashkour, M., Fontugne, M., Hatté, C. 1999. Investigations on the evolution of subsistence economy in the Qazvin Plain (Iran) from the Neolithic to the IronAge. *Antiquity* 73, 65-76.

Mavridis, F., Tankosić, Ž. 2016a. The Later Neolithic Stages in Central-Southern Greece based on the evidence from the excavations at the Agia Triada Cave, Southern Euboea. In Z. Tsirtsoni, (ed.), *The human face of radiocarbon: reassessing chronology in prehistoric Greece and Bulgaria, 5000-3000 cal BC*, Travaux de la Maison de l’Orient et de la Méditerranée No 69, Lyon, 419-436.

Mavridis, F., Tankosić, Ž. 2016b. Early Bronze Age burial deposits at the Ayia Triada cave at Karystos, Euboia: tentative interpretations. *Hesperia* 85, 207-242.

Maziar, S. 2010. Excavations at Köhné Pāsgāh Tepesi, the Araxes valley, Northwest Iran: First preliminary report. *ANES* 47, 165-193.

Maziar, S. in press 2019. Iran and the Kura-Araxes Cultural Tradition: So Near and Yet So Far*.* In J.-W. Meyer, E. Vila, R.Vallet, M. Casanova & M. Mashkour (eds), The Iranian Plateau during the Bronze Age. Development of Urbanisation, Production and Trade. Travaux de la Maison de l’Orient et Méditerranée (TMO). Lyon.

McBurney, C.B.M. 1969. The cave of Ali Tappeh and the Epipaleolithic in N.E. Iran, *Proceedings of the Prehistoric Society* 34, 385-513.

Michalopoulou, S. 2017. The Early Neolithic in West Macedonia: the contribution of the zooarchaeologial study of the sites Xirolimni-Portes and Mavropigi-Fillotsairi. Unpublished Phd Thesis, Department of History and Archaeology, University of Athens, Greece (in Greek).

Mohaseb F. A. 2012. Exploitation des animaux de l’Âge du Bronze au début de la période Islamique dans le Nord-ouest de l’Iran : L’étude archéozoologique de Haftavan Tepe. Unpublished PhD. Thesis. University of Paris I (Pantheon-Sorbonne).

Mollasalehi, H., Mashkour, M., Chaychi, A., Naderi, R. 2006. An introductory report of the archaeological investigations: the results of the stratigraphy and chronography of the pre-historic Zagheh area in Ghazvin plain in 2004. *Bastanshenasi* 2 (3), 26-46.

Negahban, E.O. 1979. A brief report on the painted building of Zagheh. Lath 7th- Early 6th Millenium B.C. *Paléorient* , 239-250.

Papathanasiou, A., Parkinson, W., Pullen, D., Galaty, M. and Karkanas P. 2018. *Neolithic Alepotrypa Cave in the Mani, Greece*. Oxbow books, Oxford.

Papayianni, K. 2012. The micromammals of Minoan Crete: Human intervention in the ecosystem of the island. In Iliopoulos, G. Meyer, C., Frey, E. Buffetaut, E, Liston, J., Osi, A. (eds.), *Proceedings of the 9th European Vertebrate Palaeontology Association Meeting, Herakleion 2011*, Paleobiodiversity and Palaeoenvironments (2012) 92, 239-248.

Papayianni, K. and Cucchi, T. 2018. The microfauna from Alepotrypa Cave. In Papathanasiou, A., Parkinson, W., Pullen, D., Galaty, M. and Karkanas P. (eds.), *Neolithic Alepotrypa Cave in the Mani, Greece*. Oxbow books.

Parés, A., Tengberg, M. 2017.Étude des pratiques d’exploitation et d’utilisation des ressources végétales du village de Khirokitia (Chypre) au Néolithique précéramique récent chypriote (VIIe-VIe millénaires av. J.‑C.). In Vigne, J.-D., Briois, F. et Tengberg, M. (eds.), *Nouvelles données sur les débuts du Néolithique à Chypre / New data on the beginning of the Neolithic in Cyprus,* Paris, Société Préhistorique de France (séances en ligne), vol. 9, 241-251.

Peltenburg, E.S., Croft, P., Jackson, A., McCartney, C., Murray, M.A. 2001. Well-established colonists: Mylouthkia 1 and the Cypro-Pre-Pottery Neolithic B. In Swiny, S. (ed.), *The earliest prehistory of Cyprus. From Colonisation to Exploitation*, vol. 2, American School of Oriental Research Institute, 61-94.

Peltenburg, E. 2003. The colonisation and settlement of Cyprus. Investigations at Kissonerga-Mylouthkia 1976-1996. Lemba Archaeological project, Cyprus, Volume III.1, *Studies in Mediterranean Archaeology* Vol. LXX:4, 84-85.

Poursat, J.-C. 2010. Malia: palace, state, city. In O. Krzyszkowska (ed.), *Cretan Offerings. Studies in Honour of Peter Warren*. London, The British School at Athens (BSA Studies, 18), 259–267.

Pulak, C. 1996. Dendrochronological dating of the Uluburun Ship. *The INA Quaterly* 23 (1), 12-14.

Pulak, C. 1998. The Uluburun shipwreck: an overview, *The International Journal of Nautical Archaeology* 27.3, 188-224.

Rosenberg, M. 1985. Report on the 1985 sondage at Eshkaft-e Gavi, *Journal of Persian Studies* *Iran* 23, 51-62.

Russell, N., Martin. L. 2005. The Çatalhöyük mammal remains. In Hodder, I. (ed.), *Inhabiting Çatalhöyük: reports from the 1995–99 seasons.* McDonald Institute Monographs. Cambridge: McDonald Institute for Archaeological Research, 35–95.

Sampson, A., Kozłowski, J.K., Kaczanowska, M., Budek, A., Nadachowski, A., Tomek, T., Miekina, B. 2009. Sarakenos Cave in Boeotia, from Palaeolithic to the Early Bronze Age. *Eurasian Prehistory* 6 (1), 199–231.

Scott, J.E., Marean, C.W. 2009. Paleolithic hominin remains from Eshkaft-e Gavi (southern Zagros Mountains, Iran): description, affinities, and evidence for butchery. *Journal of Human Evolution* 57, 248-259.

Simmons, A.H. 1999. *Faunal Extinctions in an Island Society: Pygmy Hippopotamus Hunters of Cyprus*. Kluwer Academic/Plenum Publishers. New York.

Simmons, A.H. 2013. Akrotiri-*Aetokremnos* (Cyprus) 20 years later: an assessment of its significance. In Ammermann, A.J. and Davis, T. (eds.), *Island archaeology and the origins of seafaring in the eastern Mediterranean*, Proceedings of the Wenner Gren Workshop held at Reggio Calabria o October 19-21, 2012. *Eurasian* *Prehistory* 10 (1-2), 139-156.

Smith, P.E.L. 1990. Architectural innovation and experimentation at Ganz Dareh, Iran. *World* *Archaeology* 21 (3), 323-335.

Soles, J. and Davaras, K. 1992. Excavations at Mochlos, 1989, *Hesperia* 61, 413-445

Soles, J. and Davaras, K. 2003. *Mochlos IA. Period III. Neopalatial settlement on the coast: the artisan's quarter and the farmhouse at Chalinomouri, The sites*. INSTAP Academic Press, Philadelphia.

Stevanović, M. 2002. Burned houses in the Neolithic of southeast Europe. In D. Gheorghiu (ed.) *Fire in archaeology: papers from a session held at the European Association of Archaeologists Sixth Annual Meeting in Lisbon, 2000.* British Archaeological Reports international series 1089, Oxford: Archaeopress, 55-62.

Stordeur D. 2015. Le village de Jerf El Ahmar (Syrie, 9500-8700 cal BC). Ou comment interroger l’architecture pour comprendre la société qui l’engendre, *ArchéOrient – Le Blog*(Hypotheses.org), 10 avril 2015. <http://archeorient.hypotheses.org/3900>

Stordeur, D., Helmer D., Willcox, G. 1997. Jerf el Ahmar, un nouveau site sur le moyen Euphrate, Bulletin de la Société Préhistorique Française 93, 1-4.

Stratouli, G. 2013. Νομός Καστοριάς, Δ.Δ. Αυγής, Νεολιθικός Οικισμός Αυγής [Kastoria prefecture, Neolithic Avgi settlement]. *Archaiologikon Deltion* 60:B2 (2005), 748-754.

Stratouli, G., Facorellis, Y. and Y. Maniatis 1999. Towards understanding the Late Neolithic and the Chalcolithic in the Ionian Islands, Western Greece: 14C Evidence from the “Cave of Drakaina”, Poros, Cephalonia. In J. Evin, Chr. Oberlin, J.P. Daugas and J. F. Salles (eds.), *Actes du théme Congéss International, Lyon 6-20 Avril 1998, 14C et Archéologie, Memoires de la société Préhistorique Française 26, 1999 et Supplément 1999 de la Revue* d’Archéométrie, 273-278.

Stratouli, G., Triantaphylloy, S., Bekiaris, T. and Katsikaridis, N. 2010. The manipulation of death: a burial area at the Neolithic Settlement of Avgi, NW Greece, *Documenta Praehistorica XXXVII*, 95-104.

Stratouli, G., Andreasen, N.H., Kalogiropoulou, E., Katsikaridis, N., Kloukinas, D., Κoromila, G., Margaritis, E., Bekiaris, T. 2011. ‘Houses and yards’ at the Neolithic settlement of Avgi, Kastoria: Building 5 and the adjacent open areas. *Archaeological Excavations at Macedonia and Thrace* 25, Greek Ministry of Culture and Aristoteleian Univeristy of Thessaloniki, Thessaloniki, Greece, 7-18.

Stratouli, G., Bekiaris, T., Katsikaridis, N. and Tzevelekidi, V. 2014a. Integrating the past, determining the present, and establishing the future: identification and interpretation of structured deposition at the neolithic settlement of Avgi in Kastoria, Northern Greece. In Stefani, E., Merousis, N. and Dimoula, A. (eds.), *A century of research in prehistoric Macedonia (1912-2012), International Conference Proceedings, 22-24 November 2012,* Archaeological Museum of Thessaloniki, Thessaloniki, 349-358.

Stratouli, G., Sarpaki, A., Ntinou, M., Kotjabopoulou, E., Theodoropoulou, T., Melfos, V., Andreasen, N. H., Karkanas, P. 2014b. Dialogues between bioarchaeological, geoarchaeological and archaeological data: Approaches to understanding the neolithic use of Drakaina Cave, Kephalonia Island, Western Greece. In G. Touchais, R. Laffineur & F. Rougemont (eds.), *Physis, Actes de la 14ème Rencontre égéenne internationale, Paris, INHA, 11-14 Décembre 2012*, Leuven - Liege, 23-32.

Switsur, V.R., Hall, M.A. and West, R.G. 1970. University of Cambridge Natural Radiocarbon Measurements IX, *Radiocarbon* 12, 590-598.

Switsur, V.R. and West, R.G. 1972. University of Cambridge Natural Radiocarbon Measurements X, *Radiocarbon* 14 (1), 239-246.

Tasić, N., Marić, M., Penezić, K., Filipović, D., Borojević, K., Russell, N., Reimer, P., Barclay, A., Bayliss, A., Borić, D., Gaydarska, B., Whittle, A. 2015. The end of the affair: formal chronological modelling for the top of the Neolithic tell of Vinča-Belo Brdo. *Antiquity* 89, 1064-1082. doi:10.15184/aqy.2015.101

Thiébault S. 2003. Les paysages végétaux de Chypre au Néolithique : premières données anthracologiques. In J. Guilaine et A. Le Brun (eds.), *Le Néolithique de Chypre*, actes du colloque international organisé par le département des Antiquités de Chypre et l’École française d’Athènes (Nicosie, 2001), Athènes, École française d’Athènes. *Bulletin de correspondance hellénique*, supplément 43, 221-230.

Tringham, R. 1991. Households with faces: the challenge of gender in prehistoric architectural remains. In J. Gero & M. Conkey (eds.) *Engendering archaeology: women and prehistory*, Oxford: Blackwell, 93-131.

Tringham, R. 2005. Weaving house life and death into places: a blueprint for a hypermedia narrative. In D. Bailey, A. Whittle & V. Cummings (eds.), *(un)Settling the Neolithic*, Oxford: Oxbow, 98-111.

Valamoti, S.M. 2011. Seeds for the dead? Archaeobotanical remains from Mavropigi near Kozani, site Fillotsairi. *The Archaeological Work in Upper Macedonia* 1, Aiani Kozani, 245-257.

Valamoti, S.M. 2015. Harvesting the ‘Wild’? Exploring the context of fruit and nut exploitation at Neolithic Dikili Tash, with special reference to wine. *Vegetation History and Archaeobotany* 24, 35–46.

Valla, F., Khalaily, H., Samuelian, N., Bocquentin, F., Bridault, A., Rabinovich, R. 2017. Eynan (Ain Mallaha). In Y. Enzel & O. Bar-Yosef (eds.), *Quaternary of the Levant: Environments, Climate Change and Humans,* Cambridge: Cambridge University Press, 295-302.

Vigne, J.D. 2002. Les début néolithiques de l’élevage des ongulés au Proche Orient et en Méditerranée : acquis récents et questions. In J. Guilaine (ed.), *Premiers paysans du monde: Naissance des agricultures*, Paris, France, 143-170.

Vigne, J.-D., I. Carrere, J. F. Saliege, A. Person, H. Bocherens, J. Guilaine, F. Briois. 2000. Predomestic cattle, sheep, goat and pig during the late 9th and the 8th millennium cal. BC on Cyprus: preliminary results of Shillourokambos (Parekklisha, Limassol). In M. Mashkour, A. M. Choyke, H. Buitenhuis and Poplin, F. (eds.), *Archaeozoology of the Near East IV, Proc. 4^th^ int. Symp. Archaeozoology of Southwestern Asia and adjacent areas (ASWA; Paris, June 1998).* Groningen: Archaeological Research and Consultancy (Publicaties 32), 52-75.

**Vigne, J.-D.**, Zazzo, A., Saliege, J.-F., Poplin, F., Guilaine, J., Simmons A., 2009. Pre-Neolithic wild boar management and introduction to Cyprus more than 11,400 years ago. *PNAS* 106 (38), 16.131-16.138.

**Vigne J.-D.,** Briois F., Zazzo A., Willcox G., Cucchi T., Thiébault S., Carrère I., Franel Y., Touquet R., Martin C., Moreau C., Comby C., Guilaine J., 2012. The first wave of cultivators spread to Cyprus earlier than 10,600 years ago, *PNAS* 109 (22), 8445-8449.

Vigne, J.-D., F. Briois, T. Cucchi, Y. Franel, P. Mylona, M. Tengberg, R. Touquet, J. Wattez, G. Willcox, A. Zazzo, J. Guilaine. 2017. Klimonas, a late PPNA hunter-cultivator village in Cyprus: new results. In J.D. Vigne, F. Briois, M. Tengberg (eds.), *Nouvelles données sur les débuts du Néolithique à Chypre* vol. 9 21–46, Société préhistorique française, 21-45.

Warren, P. 1976. Radiocarbon dating and calibration and the absolute chronology of late Neolithic and Early Minoan Crete, *Studi Micenei ed Egeo-Anatolici* 17, 205-219.

Weeks, L. R., Alizadeh, K., Niakan, L., Alamdari, K., Zeidi, M., and Khosrowzadeh, A. 2009. Excavations at Tol-e Nurabad. In D. T. Potts, K. Roustaei, C. A. Petrie and L. R. Weeks (eds.), *The Mamasani Archaeological Project Stage One: A Report on the First Two Seasons of the ICAR – University of Sydney Expedition to the Mamasani District, Fars Province, Iran*. BAR International Series 2044. Oxford, Archaeopress, 31-88.

Weissbrod, L., Marshall, F.B., Valla, F.R., Khalaily, H., Bar-Oz, G., Auffray, J.-C., Vigne, J.-D., Cucchi, T. 2017. Origins of house mice in ecological niches created by settled hunter-gatherers in the Levant 15,000 y ago, *PNAS*. [www.pnas.org/cgi/doi/10.1073/pnas.1619137114](http://www.pnas.org/cgi/doi/10.1073/pnas.1619137114)

Willcox, G. 1996. Evidence for plant exploitation and vegetation history from three Early Neolithic pre-pottery sites on the Euphrates (Syria). *Vegetation History and Archaeobotany* 5, 143-152.

Willcox, G. 1998. Archaeobotanical Evidence for the Beginnings of Agriculture in Southwest Asia. In Damania, A.B., Valkoun, J., Willcox, G., Qualset, C.O. (eds.), *The origins of agriculture and crop domestication, Proceedings of the Harlan Symposium (10-14 May 1997, Aleppo, Syria*), ICARDA, Aleppo, Syria, 37-52.

Zeder, M.A. and Hesse, B. 2000. The initial domestication of goats (*Capra hircus*) in the Zagros Mountains 10,000 ago, *Science* vol. 287, No 5461, 2254-2257.

Zeder, M. 2011. The origins of agriculture in the Near East, *Current Anthropology* 52 (4), 221-235.
