# Supplementary material for: Tracking the Near Eastern origins and European dispersal of the western house mouse
Source: Sci Rep. 2020 May 19;10:8276. doi: 10.1038/s41598-020-64939-9 (PMC7237409; doi:10.1038/s41598-020-64939-9)
Supplement: Supplementary file 6 — Supplementary information6. [file 41598_2020_64939_MOESM6_ESM.pdf]

Supplementary Methods S5: GMM numerical taxonomy of the archaeological samples including the results of the CVA (Figure 1) and of the machine learning identification with KNN classification (Table 1 and 2).

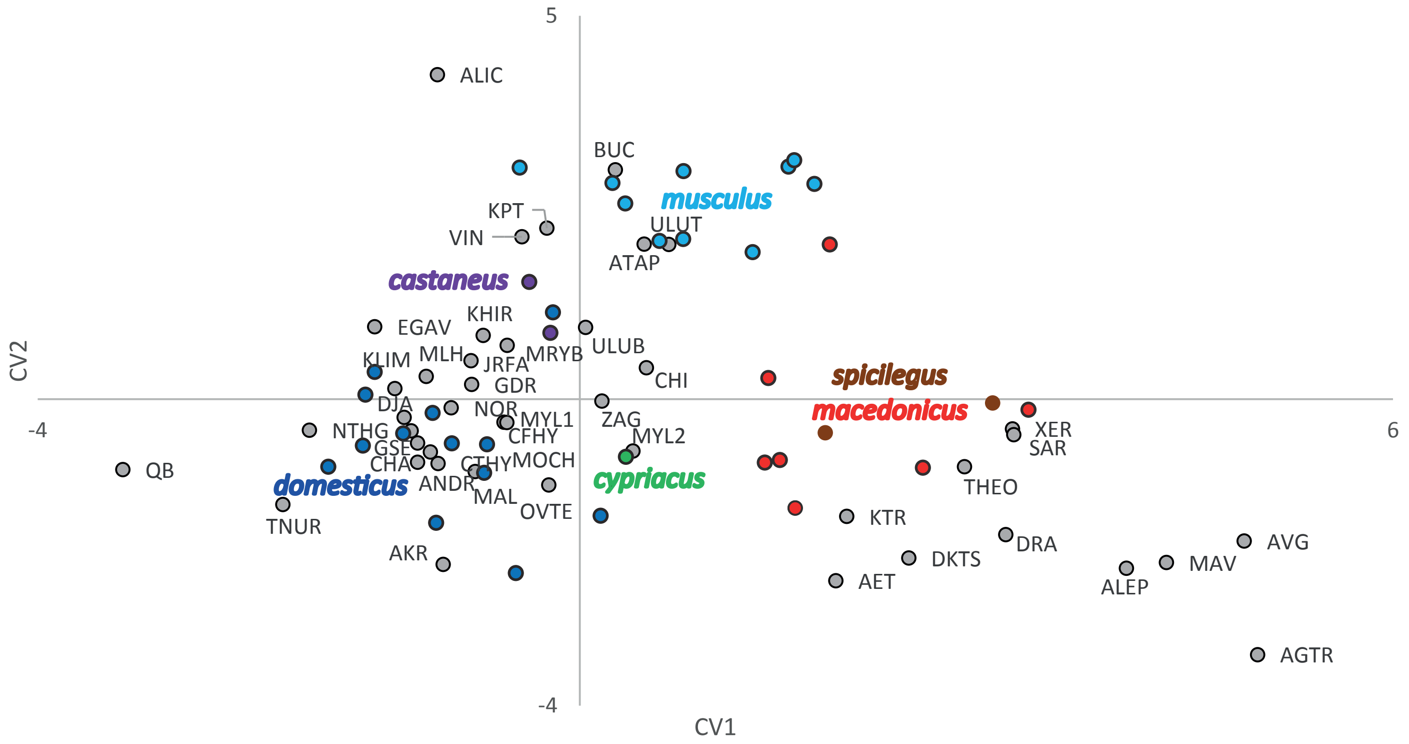

Figure 1: First two canonical axes of the CVA computed on the dental shape variation in modern wild mice and Mus musculus sub-species (dots with colors) and archaeological *Mus* sp. (dots with grey filling and acronym of the site).
Each dot represents the CVA mean scores for each modern and archaeological sample. For more informationabout the archaeological dataset see SI Table 1.

prediction	AET	AGTR	AKR	ALEP	ALIC	ANDR	ATAP	AVG	BUC	CFHY	CHA	CHI	CTHY	DJA	DKTS	DRA	EGAV	GDR	GSE	JRFA	KHIR
castaneus	0	0	0	0	0	0	0	0	0	0	0	0	0	0	0	0	0	0	0	0	0
domesticus	0	0	1	0	0	1	0	0	0	1	1	0	1	1	0	0	1	1	1	1	1
musculus	0	0	0	0	1	0	1	0	1	0	0	0	0	0	0	0	0	0	0	0	0
spici/macedo	1	1	0	1	0	0	0	1	0	0	0	1	0	0	1	1	0	0	0	0	0

prediction	KLIM	KPT	KTR	MAL	MAV	MLH	MOCH	MRYB	MYL1	MYL2	NOR	NTHG	OVTE	QB	SAR	THEO	TNUR	ULUB	ULUT	VIN	XER
castaneus	0	0	0	0	0	0	0	0	0	0	0	0	0	0	0	0	0	0	0	0	0
domesticus	1	0	0	1	0	1	1	1	1	0	1	1	1	1	0	0	1	0	0	0	0
musculus	0	1	0	0	0	0	0	0	0	0	0	0	0	0	0	0	0	1	1	1	0
spici/macedo	0	0	1	0	1	0	0	0	0	1	0	0	0	0	1	1	0	0	0	0	1

prediction	ZAG
castaneus	0
domesticus	1
musculus	0
spici/macedo	0

Table 1*: KNN* classification of the archaeological mean shapes in the discriminant morphospace (CV1 and 2) using the 4 modern taxonomic units (*musculus, domesticus, castaneus* and non commensal *cypriacus/macedonicus/spicilegus*) as class vectors and a k value of 8.

prediction	AET	AGTR	AKR	ALEP	ALIC	ANDR	ATAP	AVG	BUC	CFHY	CHA	CHI	CTHY	DJA	DKTS	DRA	EGAV	GDR	GSE	JRFA	KHIR
castaneus	0	0	0	0	0	0	0	0	0	0	0	0	0	0	0	0	0	0	0	0	0
domesticus	0	0	1	0	0	1	0	0	0	1	1	0	1	1	0	0	1	1	1	1	1
musculus	0	0	0	0	1	0	1	0	1	0	0	0	0	0	0	0	0	0	0	0	0
spici/macedo	1	1	0	1	0	0	0	1	0	0	0	1	0	0	1	1	0	0	0	0	0

prediction	KLIM	KPT	KTR	MAL	MAV	MLH	MOCH	MRYB	MYL1	MYL2	NOR	NTHG	OVTE	QB	SAR	THEO	TNUR	ULUB	ULUT	VIN	XER
castaneus	0	0	0	0	0	0	0	0	0	0	0	0	0	0	0	0	0	0	0	0	0
domesticus	1	0	0	1	0	1	1	1	1	0	1	1	1	1	0	0	1	0	0	0	0
musculus	0	1	0	0	0	0	0	0	0	0	0	0	0	0	0	0	0	1	1	1	0
spici/macedo	0	0	1	0	1	0	0	0	0	1	0	0	0	0	1	1	0	0	0	0	1

prediction	ZAG
castaneus	0
domesticus	1
musculus	0
spici/macedo	0

Table 2: *KNN* classification of the archaeological mean shapes in the discriminant morphospace (CV1 and 2) using the 4 modern taxonomic units (*musculus, domesticus, castaneus* and non commensal *cypriacus/macedonicus/spicilegus*) as class vectors and a k value of 9.
