# Supplementary material for: Tracking the Near Eastern origins and European dispersal of the western house mouse
Source: Sci Rep. 2020 May 19;10:8276. doi: 10.1038/s41598-020-64939-9 (PMC7237409; doi:10.1038/s41598-020-64939-9)
Supplement: Supplementary file 10 — Supplementary information10. [file 41598_2020_64939_MOESM10_ESM.docx]

**Supplementary Table S9: Characteristics of the three nested amplicons**

| F primer | R primer | Annealing Temperature (°C) | Amplicon length (bp) | Sequence length (bp) | Number of fixed SNPs musculus vs. domesticus |
| --- | --- | --- | --- | --- | --- |
| F529 | R593 | 58°C | 65 | 20 | 1 |
| F529 | R620 | 58°C | 92 | 45 | 2 |
| F529 | R661 | 58°C | 133 | 87 | 3 |
