# Supplementary material for: Tracking the Near Eastern origins and European dispersal of the western house mouse
Source: Sci Rep. 2020 May 19;10:8276. doi: 10.1038/s41598-020-64939-9 (PMC7237409; doi:10.1038/s41598-020-64939-9)
Supplement: Supplementary file 11 — Supplementary information11. [file 41598_2020_64939_MOESM11_ESM.docx]

**Supplementary Table S10: qPCR primers**

| Primer | Sequence (5’-3’) | Length (bp) | GC (%) | Tm (°C) | Number of mismatches with *Homo sapiens* |
| --- | --- | --- | --- | --- | --- |
| CytB-F529 | CGATTCTTCGCTTTCCACTTCATC | 24 | 45.8 | 60.5 | 5 |
| CytB-R593 | AGGAGGTGAACGATTGCTAGG | 21 | 52.4 | 59.5 | 4 |
| CytB-R620 | TTGTTTGATCCTGTTTCGTGGAG | 23 | 43.5 | 59.4 | 3 |
| CytB-R661 | GAAATGGAATTTTATCTGCATCTGAGT | 27 | 33.3 | 58.6 | 7 |
