# Supplementary material for: Tracking the Near Eastern origins and European dispersal of the western house mouse
Source: Sci Rep. 2020 May 19;10:8276. doi: 10.1038/s41598-020-64939-9 (PMC7237409; doi:10.1038/s41598-020-64939-9)
Supplement: Supplementary file 12 — Supplementary information12. [file 41598_2020_64939_MOESM12_ESM.docx]

**Supplementary Table S11 : qPCR results (in estimated copy numbers per µl of DNA extract) for samples from which sequences were obtained. PCR replicates are separated by a semicolon**

| Specimen | 63 bp amplicon  (copy number / µl) | 92 bp amplicon  (copy number / µl) | 133 bp amplicon  (copy number / µl) | GenBank ID |
| --- | --- | --- | --- | --- |
| MT2 | 1.24 | 0.4 | 0.1 | MN943333 |
| MT7 | 5.4 ; 4.3 | 1.5 ; 1.4 | 0.1 ; 0.4 | MN943334 |
| MT13 | 6.6 ; 6.2 | 2.0 ; 1.9 | 0.1 | MN943335 |
| MT16 | 1.5 ; 1.4 | 0.4 ; 0.2 | - | MN943336 |
| MT23 | 1.4 ; 1.2 | 1.0 ; 0.8 | 0.3 | MN943337 |
| MT30 | - | 0.2 ; 0.2 | - |  |
| MT35 | 15.8 ; 12.8 | 8.3 ; 7.8 | 2.4 ; 1.4 | MN943338 |
| MT36 | 18.2 ; 10.0 | 6.1 ; 9.0 | 6.1 ; 3.1 | MN943339 |
| MT37 | 41.8 ; 43 | 21.0 ; 18.8 | 5.0 ; 2.2 | MN943340 |
| MT38 | 14.4 ; 24.7 | 4.0 ; 5.9 | 1.0 ; 1.8 | MN943341 |
| MT39 | 12.9 ; 32.6 | 3.0 ; 8.9 | 1.5 ; 1.6 | MN943342 |
| MT40 | 11.6 ; 22.5 | 1.9 ; 3.8 | 0.5 ; 1.24 | MN943343 |
| MT41 | 50.7 ; 47.4 | 20.0 ; 17.7 | 0.7 ; 4.6 | MN943344 |
| MT42 | 24.3 | 9.9 ; 13.2 | 3.0 ; 4.6 | MN943345 |
| MT43 | 3.6 ; 1.9 | 1.4 ; 0.7 | 0.4 | MN943346 |
